# Identification of a Novel Glycolysis-Related LncRNA Signature for Predicting Overall Survival in Patients With Bladder Cancer

**DOI:** 10.3389/fgene.2021.720421

**Published:** 2021-08-19

**Authors:** Zhenming Zheng, Cong Lai, Wenshuang Li, Caixia Zhang, Kaiqun Ma, Yousheng Yao

**Affiliations:** ^1^Guangdong Provincial Key Laboratory of Malignant Tumor Epigenetics and Gene Regulation, Sun Yat-sen Memorial Hospital, Sun Yat-sen University, Guangzhou, China; ^2^Department of Urology, Sun Yat-sen Memorial Hospital, Sun Yat-sen University, Guangzhou, China; ^3^Guangdong Provincial Clinical Research Center for Urological Diseases, Guangzhou, China

**Keywords:** bladder cancer, glycolysis, lncRNAs, prognosis, signature

## Abstract

**Background:**

Both lncRNAs and glycolysis are considered to be key influencing factors in the progression of bladder cancer (BCa). Studies have shown that glycolysis-related lncRNAs are an important factor affecting the overall survival and prognosis of patients with bladder cancer. In this study, a prognostic model of BCa patients was constructed based on glycolysis-related lncRNAs to provide a point of reference for clinical diagnosis and treatment decisions.

**Methods:**

The transcriptome, clinical data, and glycolysis-related pathway gene sets of BCa patients were obtained from The Cancer Genome Atlas (TCGA) database and the Gene Set Enrichment Analysis (GSEA) official website. Next, differentially expressed glycolysis-related lncRNAs were screened out, glycolysis-related lncRNAs with prognostic significance were identified through LASSO regression analysis, and a risk scoring model was constructed through multivariate Cox regression analysis. Then, based on the median of the risk scores, all BCa patients were divided into either a high-risk or low-risk group. Kaplan-Meier (KM) survival analysis and the receiver operating characteristic (ROC) curve were used to evaluate the predictive power of the model. A nomogram prognostic model was then constructed based on clinical indicators and risk scores. A calibration chart, clinical decision curve, and ROC curve analysis were used to evaluate the predictive performance of the model, and the risk score of the prognostic model was verified using the TCGA data set. Finally, Gene Set Enrichment Analysis (GSEA) was performed on glycolysis-related lncRNAs.

**Results:**

A total of 59 differentially expressed glycolysis-related lncRNAs were obtained from 411 bladder tumor tissues and 19 pericarcinomatous tissues, and 9 of those glycolysis-related lncRNAs (AC099850.3, AL589843.1, MAFG-DT, AC011503.2, NR2F1-AS1, AC078778.1, ZNF667-AS1, MNX1-AS1, and AC105942.1) were found to have prognostic significance. A signature was then constructed for predicting survival in BCa based on those 9 glycolysis-related lncRNAs. ROC curve analysis and a nomogram verified the accuracy of the signature.

**Conclusion:**

Through this study, a novel prognostic prediction model for BCa was established based on 9 glycolysis-related lncRNAs that could effectively distinguish high-risk and low-risk BCa patients, and also provide a new point of reference for clinicians to make individualized treatment and review plans for patients with different levels of risk.

## Introduction

Bladder cancer (BCa) is one of the most prevalent malignancies of the urinary system, according to statistics from 185 countries worldwide, in 2020, it ranked 9th in incidence and 13th in mortality (Sung et al., 2021). BCa is a heterogeneous disease and can be divided into two clinical types depending on whether it infiltrates the muscle layer: 25% of cases are muscle invasive BCa (MIBC), and 75% are non-muscle invasive BCa (NMIBC) (Cumberbatch et al., 2018). NMIBC has a low mortality rate, while 70% of these tumors will recur and 15% will progress in stage and grade. In contrast, the long-term survival rate of MIBC is unsatisfactory because it is prone to develop distant metastasis (Knowles and Hurst, 2015). Recent studies suggested that the poor prognosis of MIBC may also be related to the different molecular mechanisms of its occurrence and progression; some scholars even believed that MIBC and NMIBC were not the same kind of disease (Gui et al., 2011). With the increasing in-depth study of molecular biological mechanisms and the rapid development of gene detection technology, molecular typing and prognostic evaluation of BCa by genetic testing is becoming a new diagnostic and therapeutic target.

Glycolysis is a metabolic pathway that provides energy to the body. Changes in the glycolytic state may signify the presence of cancer. One study by Otto Warburg found that the glycolysis of tumor cells produced more lactic acid and less ATP, which resulted in tissue hypoxia, even in an environment with a high oxygen content. This phenomenon is known as the “Warburg effect” (Warburg, 1956). Enhanced or abnormally activated glycolysis promoted tumor progression, which has been proven in breast cancer, colorectal cancer, liver cancer, etc. (Chen et al., 2019a; Weng et al., 2020; Zhang et al., 2020). In BCa, an increase of the Warburg effect has been proven to contribute to the aggressiveness of BCa tumor cells and accelerated the proliferation rate of BCa cells (Ritterson Lew et al., 2015). Therefore, changes in glycolytic status may be an emerging marker of malignancy and a potential prognostic target for patients with BCa.

LncRNAs are a class of nucleotides with a transcriptional length of more than 200 nucleotides. Although lncRNAs do not possess protein-coding ability, they can regulate gene expression through multiple layers and pathways, thus affecting the occurrence and progression of tumors, and can be used as markers for tumor diagnosis and prognosis (Evans et al., 2016). There are more than 50,000 lncRNAs in the human genome, and they can play different roles in different tumors, acting as tumor-suppressor or tumor-promoter genes (Derrien et al., 2012). To effectively predict the prognosis of patients with BCa, the sensitivity and specificity of a single lncRNA was not sufficient; it was necessary to combine the high or low expression of multiple lncRNAs to have stronger diagnostic value.

Hyperglycolysis is a common phenomenon in the rapid proliferation of tumor cells and is also a marker of tumorigenesis. Recently, increasing amounts of data have shown that lncRNAs were a crucial regulator of glycolysis, and some studies have attempted to prevent tumor progression by inhibiting the activity of key enzymes in the tumor glycolysis pathway (Yang et al., 2014; Chen et al., 2019a; Liao et al., 2019; Wang et al., 2019a). The functional study of glycolysis-related lncRNAs has found that different lncRNAs could promote glycolysis in various ways, which in turn promoted tumorigenesis, progression, and metastasis. For example, lncRNA-SNHG7 regulated by c-MYC could increase glycolysis by inhibiting the expression of miR-34a-5p, thus promoting the progression of breast cancer (Zhang et al., 2019), while the lncRNA LINRIS stabilized IGF2BP2 and promoted aerobic glycolysis in colorectal cancer (Wang et al., 2019b).

The functional study of glycolysis-related lncRNAs in bladder cancer has found that lncRNA-SLC16A1-AS1 could be used as a target of E2F1 and a co-activator to induce metabolic reprogramming in the progression of bladder cancer (Logotheti et al., 2020), and lncRNA UCA1 up-regulated hexokinase 2 in bladder tumor cells to promote glycolysis through the mTOR–STAT3/microRNA143 pathway (Li et al., 2014). Prognostic models constructed by differentially expressed glycolysis-related lncRNAs have been proven to have good predictive performance in gastrointestinal tumors, breast cancer, gliomas, and other tumors. In bladder cancer, there is currently no known glycolysis-related lncRNA prognostic model.

This study aimed to clarify the relationship between glycolysis-related lncRNAs and the prognosis of BCa. First, a total of 9 glycolysis-related lncRNAs were identified to have prognostic significance. Then, based on these 9 glycolysis-related lncRNAs, a novel prognostic prediction model for BCa was constructed and verified, which proved that the model possessed good predictive power. Finally, gene function enrichment analysis was performed on the differentially expressed glycolysis-related lncRNAs identified through this study to explore their potential functions in the progression of BCa.

## Materials and Methods

### Sample Sources and Processing

Transcriptome sequencing data and clinical data of BCa patients were downloaded from The Cancer Genome Atlas (TCGA) database^1^, and gene sets of glycolysis related functions and glycolysis related pathways were obtained from the Gene Set Enrichment Analysis (GSEA) official website^2^. The “edgeR” package of R software was used to normalize the entire data set, set | log2FC | > 1 and FDR < 0.05 as the threshold to construct the volcano map, and obtain the tumor tissue and pericarcinomatous differentially expressed glycolysis-related genes and lncRNA. The correlation between the lncRNAs and glycolysis-related genes was calculated using Pearson correlation. If the correlation coefficient of lncRNA | R2 | > 0.3 and *P* < 0.05, it was considered to be related to glycolysis and used for further analysis.

### Construction of the Risk Score Model

Univariate Cox regression and Least Absolute Shrinkage and Selection Operator (LASSO) regression analysis were used to screen out 9 lncRNAs with prognostic significance. Genes with *P* < 0.05 were considered to be independent glycolysis-related lncRNAs with prognostic significance. Multivariate Cox regression was used to calculate their respective coefficients (βI). Then, the formula of risk scoring model composed of βI and lncRNA expression level (EXPI) was obtained as follows: Risk score = ∑i=19(βEi*xpi). Then the risk score formula was used to calculate the risk score for each BCa patient. Subsequently, the median of the risk score was set as the cutoff value, and all eligible patients with bladder cancer were divided into high-risk and low-risk groups. Next, Kaplan-Meier survival analysis was performed to compare the differences in Overall Survival (OS) between the two groups of BCa patients. Univariate Cox regression and multivariate Cox regression analysis were used to evaluate whether risk score, age, gender, and TNM were independent prognostic factors for BCa. Clinical survival analysis was used to evaluate the predictive power of the risk scoring model for different clinical subgroups. ROC curve analysis was used to evaluate the efficiency of the prognostic model. These results were also verified by the validation data.

### Establishment and Evaluation of the Prognostic Model

A nomogram was then constructed to obtain the 3-year and 5-year OS of BCa patients. This nomogram included the results of multivariate Cox regression, risk scores, and clinical information such as gender, age, and TNM stage. The calibration chart generated by the “rms” package of the R software verified the predictive performance of the nomogram, and the ROC curve analysis was used to evaluate the accuracy of the nomogram. Then, a decision curve analysis (DCA) was performed to verify the predictive effect of the prognostic model.

### Gene Set Enrichment Analysis

Kyoto Encyclopedia of Genes and Genomes (KEGG) analysis was performed on the differentially expressed genes of BCa patients between the high-risk and low-risk groups. By analyzing the tumor signaling pathways and tumor progression processes involved in the differentially expressed genes, their possible roles and mechanisms in the occurrence and progression of BCa was further explored.

## Results

### Differentially Expressed Glycolysis-Related Genes and LncRNAs in Bladder Cancer

**FIGURE 1:**
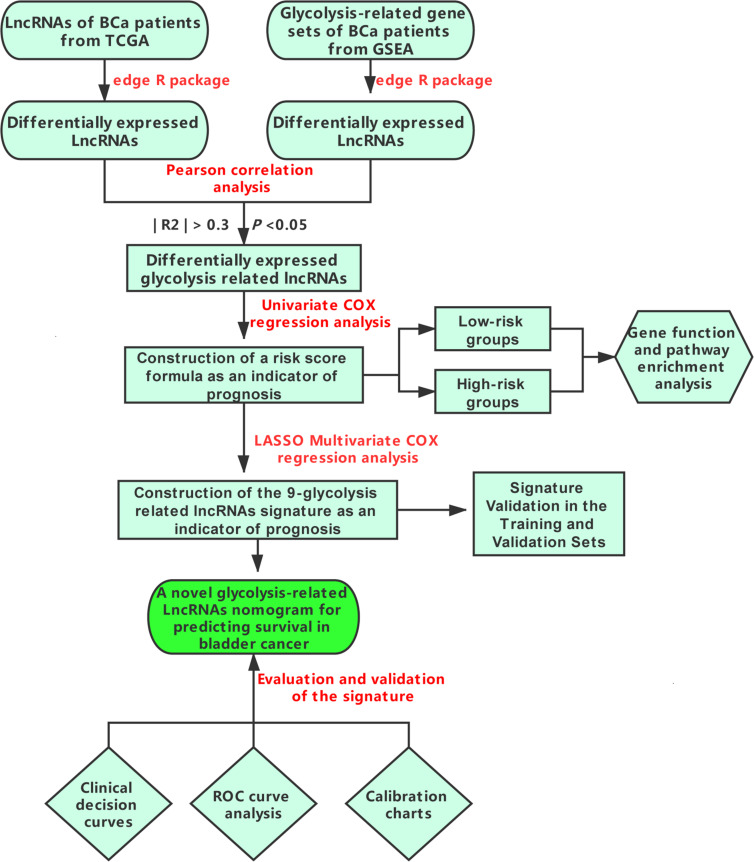
Flow chart of key steps during the process.

**FIGURE 2:**
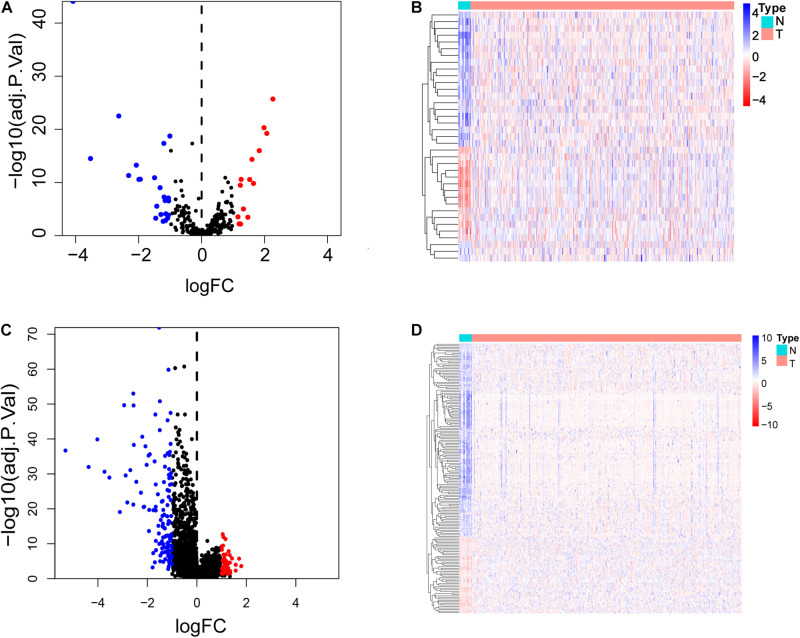
Differentially expressed glycolysis-related genes and lncRNAs in bladder cancer compared to pericarcinomatous tissues. **(A,B)** Volcano plot and heat map of glycolysis-related genes differentially expressed in bladder cancer tissue and pericarcinomatous tissue. **(C,D)** Volcano plot and heat map of lncRNAs differentially expressed in bladder cancer tissues and pericarcinomatous tissue.

The flow chart in Figure 1 shows the general step-by-step process of this study. First, the gene expression data, lncRNA sequencing data and clinical data of the corresponding BCa patients (411 tumor tissues, 19 pericarcinomatous tissues) were downloaded from the TCGA database, and glycolysis-related gene sets (GLYCOLYTIC_PRCOESS, HALLMARK_GLYCOLYSIS, REACTOME_GLYCOLYSIS, GLYCOLYSIS_GLUCONEOGENE SIS, and BIOCARTA_GLYCOLYSIS_PATHWAY) were downloaded from the GSEA database. The “edgeR” package of R software was used to normalize the entire data set, Set | log2FC | > 1 and false discovery rate (FDR) < 0.05 as the threshold to obtain glycolysis-related genes and lncRNAs that were differentially expressed between tumors and pericarcinomatous tissues (Figure 2).

**FIGURE 3:**
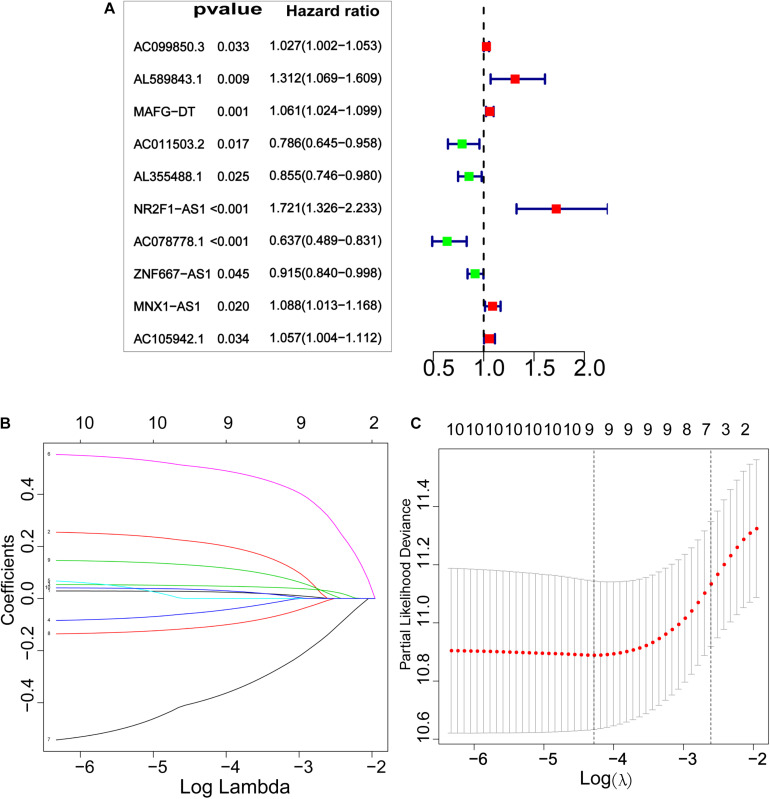
Screening glycolysis-related lncRNAs with prognostic value. **(A)** Risk ratio Forest plot identified 10 glycolysis-related lncRNAs with prognostic significance (*P* < 0.05). **(B,C)** LASSO regression further screened out 9 glycolysis-related lncRNAs that were significantly related to prognosis.

Next, Pearson correlation analysis was used to analyze the correlation between the differentially expressed glycolysis-related genes and lncRNAs, and to set correlation coefficients | R2 | > 0.3 and *P* < 0.05 for lncRNAs that were considered to be related to glycolysis. A total of 59 glycolysis-related lncRNAs were identified as differentially expressed in BCa tumors and pericarcinomatous tissues (Supplementary Table 1). Subsequently, univariate Cox regression analysis was performed, through which 10 glycolysis-related lncRNAs related to the prognosis of BCa were identified (Figure 3A). Lastly, 9 key glycolysis-related lncRNAs associated with BCa prognosis were further screened by LASSO regression (Figures 3B,C).

### Construction of a Risk Scoring Model for 9 Glycolysis-Related LncRNAs

**FIGURE 4:**
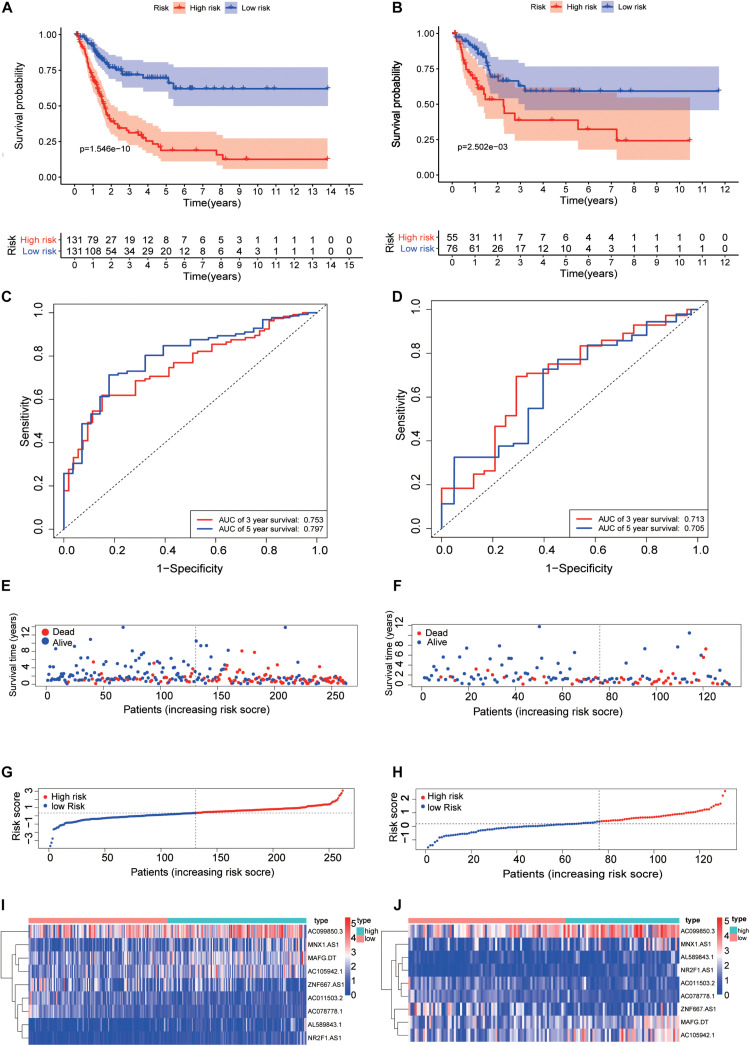
Construction of risk score model. Kaplan-Meier survival analysis showed that the Overall Survival (OS) of patients in the high-risk training set **(A)** and validation set **(B)** was significantly lower than that in the low-risk group. The ROC curve of the training set **(C)** showed that the AUCs of the 3-year and 5-year OS were 0.753 and 0.797, respectively, while the validation set **(D)** were 0.713 and 0.705, respectively. Survival rate and survival status of bladder cancer patients in the training set **(E)** and validation set **(F)**. The distribution of 9 glycolysis-related lncRNAs risk scores for each patient in the training set **(G)** and validation set **(H)**. Heatmap of 9 glycolysis-related lncRNAs in the low-risk group and the high-risk group in the training set **(I)** and validation set **(J)**.

The data of bladder cancer patients with a follow-up time of more than 30 days was selected to construct a risk scoring model, where their clinical parameters and pathological stages were showed in Supplementary Table 2. Patients were divided into training set and validation set at a ratio of 2:1. The risk score formula (Risk score = ∑i=19(βiE*xpi)) was used to calculate the risk scores of all BCa patients. The median of the risk score was set as the cutoff value, and all eligible patients with BCa were divided into either high-risk or low-risk groups. Results of Kaplan-Meier survival analysis showed that the Overall Survival (OS) of the high-risk group of BCa patients was significantly lower than that of the low-risk group, both in the training set and the validation set (*P* = 1.546e-10, Figures 4A,B). The glycolysis-related lncRNAs AC011503.2, AC078778.1, and ZNF667-AS1 were expressed at a lesser extent in tumor tissues of patients with BCa and were thus considered to be protective factors for the prognosis of bladder cancer, while glycolysis-related lncRNA AC099850.3, AL589843.1, MAFG -DT, NR2F1-AS1, MNX1-AS1, and AC105942.1 were highly expressed in tumor tissues and were therefore considered to be risk factors for the prognosis of BCa. ROC curve analysis proved that the model has a good predictive performance (3-year AUC = 0.753, 5-year AUC = 0.797) (Figure 4C). We evaluated the survival times of patients in the high- and low-risk groups and found that mortality rates for patients with high-risk scores were higher than those with low-risk scores (Figures 4E,G). Heatmap analysis was performed to reveal the expression profiles of the 9 glycolysis-related lncRNAs in high-risk and low-risk groups (Figure 4I). These results were then validated using validation set data (Figures 4B,D,F,H,J).

### Construction and Evaluation of Prognostic Model of 9 Glycolysis-Related LncRNAs

**FIGURE 5:**
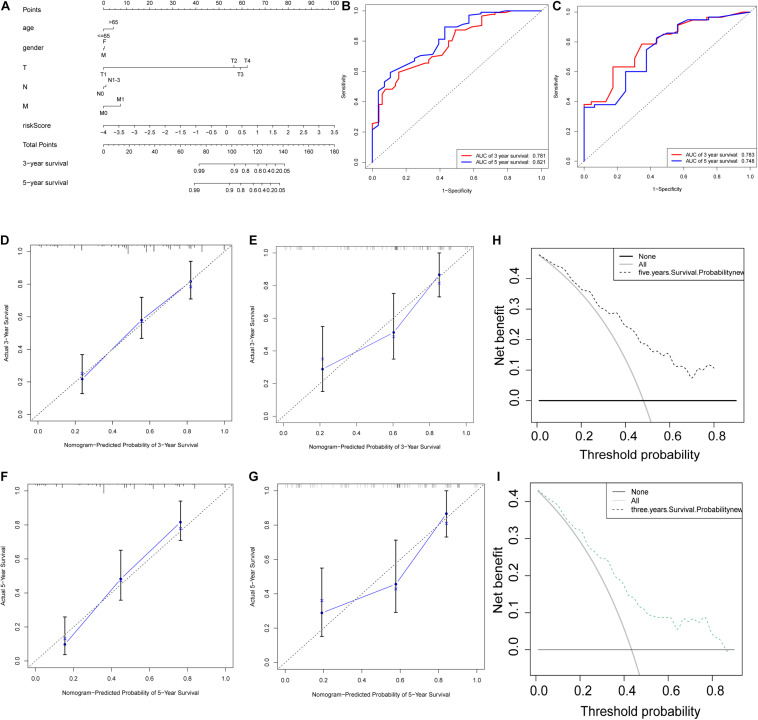
Establishment and evaluation of the nomogram. **(A)** Nomogram for predicting the 3-year and 5-year survival rates of bladder cancer patients. **(B,C)** ROC curve analysis results showed that the AUC of the training set **(B)** for 3-year OS and 5-year OS were 0.781 and 0.821, respectively, and those in the validation set **(C)** were 0.783 and 0.748, respectively. **(D–G)** The calibration curves for the 3-year and 5-year OS of the nomogram in the training set **(D,F)** and validation set **(E,G)**. **(H,I)** The clinical decision curve for the 3-year OS **(H)** and 5-year OS **(I)** of the nomogram.

**FIGURE 6:**
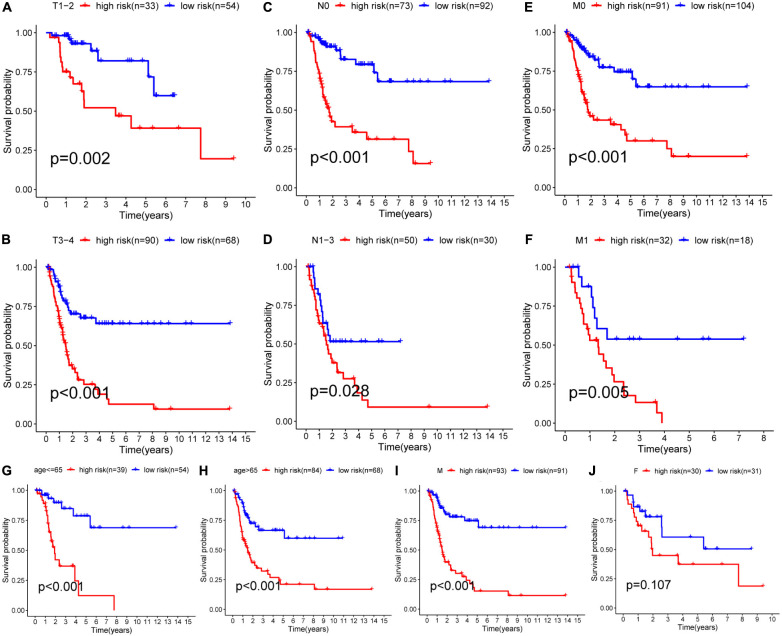
The results of subgroup analysis. Analysis showed that the OS of bladder cancer patients in the high-risk group was significantly lower than that in the low-risk group based on age and TNM stage (*P* < 0.05). **(A–I)** In male patients, the OS in the high-risk group was also significantly shorter than that in the low-risk group (*P* < 0.001), while no significant difference was shown in female patients (*P* = 0.107) **(J)**, possibly due to the relatively small number of cases.

A nomogram was then constructed to obtain the 3-year and 5-year OS of BCa patients (Figure 5A). This nomogram included the results of multivariate Cox regression, risk scores, and clinical information such as gender, age, and TNM stage. The calibration chart (Figures 5D,F) generated by using the “rms” package of the R software helped verify the performance of the nomogram. Next, the ROC curve (Figure 5B) was used to obtain the area under the curve (3-year AUC = 0.781, 5-year AUC = 0.821) to evaluate the accuracy of the nomogram. These results further proved that the prognostic signatures of the 9 glycolysis-related lncRNAs were independent prognostic factors for bladder cancer. Subsequently, the C index was calculated (training set 0.79, validation set 0.724), and Decision Curve Analysis (Figures 5H,I) was conducted to verify the predictive effect of the prognostic model. Finally, the validation set data was used to verify these results (Figures 5C,E,G). Subgroup analysis results (Figure 6) showed that the model had good predictive efficacy in all age groups, in the T1-T2 and T3-T4 stages, with or without lymph node metastasis, and with or without distant metastasis (*P* < 0.05) (Figures 6A–I). There was no significant difference observed in the female group (*P* = 0.107) (Figure 6J). Possibly because of the small number of cases in the group.

### Gene Function and Pathway Enrichment Analysis of 9 Glycolysis Related LncRNAs Signatures

**FIGURE 7:**
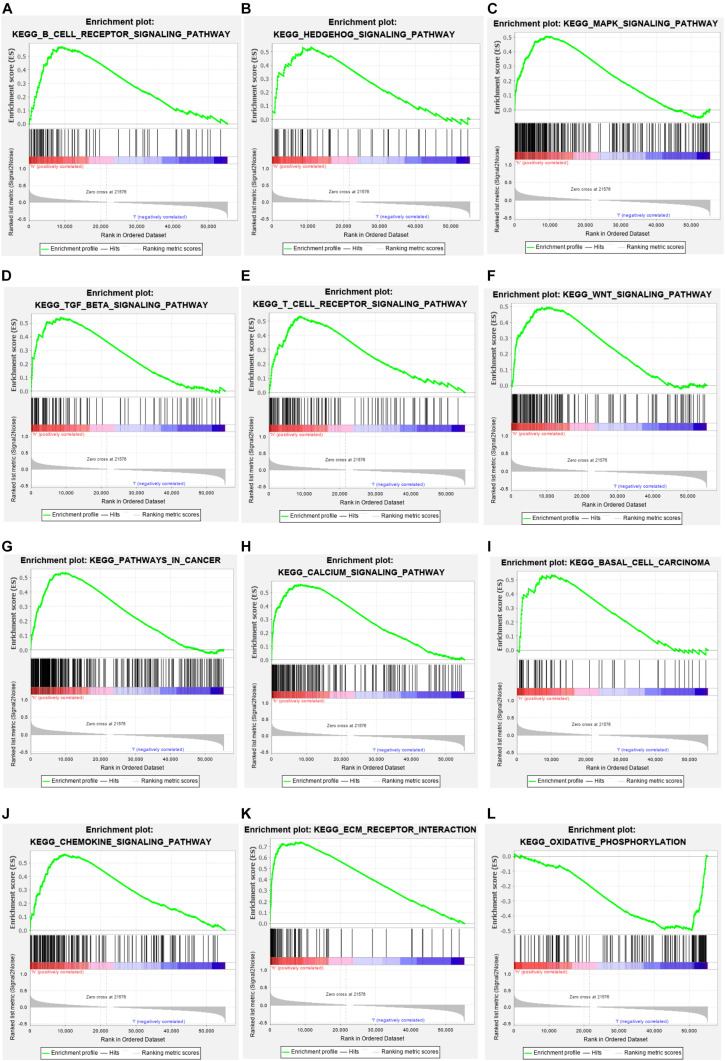
Enrichment analysis. **(A–L)** KEGG pathway analysis indicates that these glycolysis related lncRNAs were involved in the B cell and T cell receptor signaling pathway, Hedgehog signaling pathway, MAPK signaling pathway, TGF-β signaling pathway, chemokine signaling pathway, EMC receptor interaction, and oxidative phosphorylation processes.

Kyoto Encyclopedia of Genes and Genomes enrichment analysis was performed to explore the possible roles and mechanisms of the differentially expressed glycolysis-related lncRNAs in the occurrence and progression of tumors in the high-risk group and the low-risk group of BCa patients, Analysis results (Figures 7A–L) showed that these glycolysis-related lncRNAs were involved in multiple signaling pathways, such as: B-cell receptor, T-cell receptor, Hedgehog, MAPK, WNT, Calcium, and Chemokine. They were also involved in oxidative phosphorylation, ECM receptor interaction, and the development of basal cell carcinoma. These findings may help researchers determine the direction of further in-depth research in order to continue studying the mechanism of how glycolysis-related lncRNAs affects the progression of BCa.

## Discussion

Accurately predicting the prognosis of BCa patients is of great concern for clinicians in order to develop personalized treatment and review plans. For patients with a high risk of recurrence, we must be more cautious in the treatment strategy of bladder preservation, and for low-risk patients, we need to preserve the bladder as much as possible for the patients to help improve their postoperative quality of life. To determine whether a BCa patient was of a high-risk type, current clinical practice was mainly based on TNM staging, postoperative pathological diagnosis, and molecular classification, but there was no quantitative risk score or predictive model. Prognostic models constructed by differentially expressed glycolysis-related lncRNAs have been proven to have good predictive performance in gastrointestinal tumors, breast cancer, gliomas, and other tumors. Ho et al. (2021) constructed a glycolysis-related lncRNA prognosis model that could identify a subgroup of patients with different prognosis and different degrees of immune infiltration. In bladder cancer, there is currently no known glycolysis-related lncRNA prognostic model.

This prediction model was established based on the following theoretical basis and previous research results: First, an increasing amount of evidence shows that changes in glucose metabolism are a sign of tumorigenesis. Some research has pointed out that the aerobic glycolysis of bladder cancer cells is more active than that of normal urothelial cells, and the increase in aerobic glycolysis is beneficial to the proliferation, invasion, and metastasis of bladder tumor cells (Berrondo et al., 2016). Other studies have found that the activation of the Warburg effect was positively correlated with the degree of tumor malignancy (Agnihotri and Zadeh, 2016). Secondly, previous studies have confirmed that lncRNAs were also involved in glycometabolic reprogramming of tumor cells in BCa. Different lncRNAs play different roles in the glycolysis process of bladder tumor cells. Some lncRNAs accelerate tumor progression by promoting glycolysis, and some of them inhibit glycolysis to suppress cancer. For instance, Li et al. (2014) found that the lncRNA UCA1 could promote glycolysis by upregulation of HK2 through the mTOR-STAT3/miRNA and miR-143 pathways. Hu et al. (2017) found that the lncRNA CASC 8 may act as a tumor suppressor by reducing glycolysis in bladder tumor cells.

In this study, 9 glycolysis-related lncRNAs were screened in relation to the prognosis of BCa patients (AC099850.3, AL589843.1, MAFG-DT, AC011503.2, NR2F1-AS1, AC078778.1, ZNF667-AS1, MNX1-AS1, and AC105942.1). Among them, glycolysis-related lncRNA AC011503.2, AC078778.1, and ZNF667-AS1 were protective factors for the prognosis of BCa, and glycolysis-related lncRNAs AC099850.3, AL589843.1, MAFG-DT, NR2F1-AS1, MNX1-AS1, and AC105942.1 were risk factors for the prognosis of bladder cancer. MNX1-AS1 regulates the expression of RAB1A in bladder tumor cells by competitively binding with miR-218-5p, thereby promoting the proliferation, migration, invasion, and epithelial-mesenchymal transition of bladder tumor cells, which contributes to the growth and metastasis of bladder cancer cells (Wang et al., 2020). Multivariate Cox analysis was used to calculate the regression coefficient and construct the prognostic model. According to the risk score, the median was used to divide the bladder cancer patients into high-risk and low-risk groups. Analysis showed that the OS in the low-risk group was longer than that in the high-risk group. According to the results of multivariate Cox regression, a histogram was established, including age, sex, TNM stage, and risk score. The prediction performance of the model was verified by both a calibration diagram and decision curve analysis, and the results demonstrated that the model had good predictive performance.

In terms of GSEA functional enrichment analysis, it was found that these glycolysis related lncRNAs were significantly enriched for the regulation of multiple processes, such as the B cell and T cell receptor signaling pathway, Hedgehog signaling pathway, MAPK signaling pathway, TGF-β signaling pathway, chemokine signaling pathway, EMC receptor interaction, and oxidative phosphorylation processes. Li et al. (2016) found that GALNT1-mediated glycosylation and activation of Sonic Hedgehog signaling were involved in tumor-initiation and self-renewal of bladder cancer stem cells (Li et al., 2016). In another study, Teng Hu et al. found that RSPO3 promoted the aggressiveness of bladder cancer via the Wnt/β-catenin and Hedgehog signaling pathways (Chen et al., 2019b). Transcriptional regulatory factor YAP was overexpressed in bladder cancer tissue and promoted the spread of bladder cancer by affecting the MAPK pathway (Qiu et al., 2020). All of these prior results further confirmed that the glycolysis associated lncRNAs screened in the present study play an important role in the occurrence and progression of BCa.

It should be pointed out that this study has some limitations: First, this study was a retrospective study of patient data from the TCGA database, which may lead to selection bias. Secondly, the patient information obtained for this study lacks longer follow-up time; therefore, the predictive model still needs to be further verified in large-scale prospective clinical trials.

## Conclusion

This study established a novel prognostic model of BCa based on 9 glycolysis-related lncRNAs, which can effectively distinguish high-risk and low-risk bladder cancer patients from a new perspective. It can also provide a new point of reference of ideas for clinicians to formulate individualized treatment and review plans for patients with different levels of risk. This study also found that some new glycolysis-related lncRNAs were related to the prognosis of bladder cancer, which points out the direction for the next step of verification experiments and exploration of mechanisms.

## Data Availability Statement

The original contributions presented in the study are included in the article/Supplementary Material, further inquiries can be directed to the corresponding author.

## Author Contributions

YY designed the study and took overall control of the manuscript. ZZ analyzed and interpreted the data and drafted the manuscript. CL performed the data analysis and helped write the manuscript. WL collected relevant references, sorted out the data, and helped draft the manuscript. KM and CZ was involved in collecting the data and assisting with manuscript revision. All authors agreed to be accountable for the content of the work.

## Conflict of Interest

The authors declare that the research was conducted in the absence of any commercial or financial relationships that could be construed as a potential conflict of interest. The reviewer XZ declared a shared affiliation with the authors to the handling editor at the time of the review.

## Publisher’s Note

All claims expressed in this article are solely those of the authors and do not necessarily represent those of their affiliated organizations, or those of the publisher, the editors and the reviewers. Any product that may be evaluated in this article, or claim that may be made by its manufacturer, is not guaranteed or endorsed by the publisher.

## Footnotes

1
https://cancergenome.nih.gov/

2
www.gsea-msigdb.org/

## References

[B1] Agnihotri S. Zadeh G. (2016). Metabolic reprogramming in glioblastoma: the influence of cancer metabolism on epigenetics and unanswered questions. *Neuro. Oncol.* 18 160–172. 10.1093/neuonc/nov125 26180081 PMC4724176

[B2] Berrondo C. Flax J. Kucherov V. Siebert A. Osinski T. Rosenberg A. (2016). Expression of the long non-coding RNA HOTAIR correlates with disease progression in bladder cancer and is contained in bladder cancer patient urinary exosomes. *PloS One* 11:e0147236. 10.1371/journal.pone.0147236 26800519 PMC4723257

[B3] Chen F. Chen J. Yang L. Liu J. Zhang X. Zhang Y. (2019a). Extracellular vesicle-packaged HIF-1α-stabilizing lncRNA from tumour-associated macrophages regulates aerobic glycolysis of breast cancer cells. *Nat. Cell Biol.* 21 498–510. 10.1038/s41556-019-0299-0 30936474

[B4] Chen Z. Zhou L. Chen L. Xiong M. Kazobinka G. Pang Z. (2019b). RSPO3 promotes the aggressiveness of bladder cancer via Wnt/β-catenin and Hedgehog signaling pathways. *Carcinogenesis* 40 360–369. 10.1093/carcin/bgy140 30329043

[B5] Cumberbatch M. Jubber I. Black P. Esperto F. Figueroa J. Kamat A. (2018). Epidemiology of bladder cancer: a systematic review and contemporary update of risk factors in 2018. *Euro. Urol.* 74 784–795. 10.1016/j.eururo.2018.09.001 30268659

[B6] Derrien T. Johnson R. Bussotti G. Tanzer A. Djebali S. Tilgner H. (2012). The GENCODE v7 catalog of human long noncoding RNAs: analysis of their gene structure, evolution, and expression. *Genome Res.* 22 1775–1789. 10.1101/gr.132159.111 22955988 PMC3431493

[B7] Evans J. Feng F. Chinnaiyan A. (2016). The bright side of dark matter: lncRNAs in cancer. *J. Clin. Invest.* 126 2775–2782. 10.1172/jci84421 27479746 PMC4966302

[B8] Gui Y. Guo G. Huang Y. Hu X. Tang A. Gao S. (2011). Frequent mutations of chromatin remodeling genes in transitional cell carcinoma of the bladder. *Nat. Genet.* 43 875–878. 10.1038/ng.907 21822268 PMC5373841

[B9] Ho K. Huang T. Shih C. Lee Y. Liu A. Chen P. (2021). Glycolysis-associated lncRNAs identify a subgroup of cancer patients with poor prognoses and a high-infiltration immune microenvironment. *BMC Med.* 19:59. 10.1186/s12916-021-01925-6 33627136 PMC7905662

[B10] Hu R. Zhong P. Xiong L. Duan L. (2017). Long noncoding RNA cancer susceptibility candidate 8 suppresses the proliferation of bladder cancer cells via regulating glycolysis. *DNA Cell Biol.* 36 767–774. 10.1089/dna.2017.3785 28759252

[B11] Knowles M. Hurst C. (2015). Molecular biology of bladder cancer: new insights into pathogenesis and clinical diversity. *Nat. Rev. Cancer* 15 25–41. 10.1038/nrc3817 25533674

[B12] Li C. Du Y. Yang Z. He L. Wang Y. Hao L. (2016). GALNT1-mediated glycosylation and activation of sonic hedgehog signaling maintains the self-renewal and tumor-initiating capacity of bladder cancer stem cells. *Cancer Res.* 76 1273–1283. 10.1158/0008-5472.Can-15-2309 26676748

[B13] Li Z. Li X. Wu S. Xue M. Chen W. (2014). Long non-coding RNA UCA1 promotes glycolysis by upregulating hexokinase 2 through the mTOR-STAT3/microRNA143 pathway. *Cancer Sci.* 105 951–955. 10.1111/cas.12461 24890811 PMC4317864

[B14] Liao M. Liao W. Xu N. Li B. Liu F. Zhang S. (2019). LncRNA EPB41L4A-AS1 regulates glycolysis and glutaminolysis by mediating nucleolar translocation of HDAC2. *EBioMedicine* 41 200–213. 10.1016/j.ebiom.2019.01.035 30796006 PMC6444057

[B15] Logotheti S. Marquardt S. Gupta S. Richter C. Edelhäuser B. Engelmann D. (2020). LncRNA-SLC16A1-AS1 induces metabolic reprogramming during Bladder Cancer progression as target and co-activator of E2F1. *Theranostics* 10 9620–9643. 10.7150/thno.44176 32863950 PMC7449907

[B16] Qiu D. Zhu Y. Cong Z. (2020). YAP Triggers Bladder Cancer Proliferation by Affecting the MAPK Pathway. *Cancer Manag. Res.* 12 12205–12214. 10.2147/cmar.S273442 33273857 PMC7707444

[B17] Ritterson Lew C. Guin S. Theodorescu D. (2015). Targeting glycogen metabolism in bladder cancer. *Nat. Rev. Urol.* 12 383–391. 10.1038/nrurol.2015.111 26032551 PMC4678000

[B18] Sung H. Ferlay J. Siegel R. Laversanne M. Soerjomataram I. Jemal A. (2021). Global cancer statistics 2020: GLOBOCAN estimates of incidence and mortality worldwide for 36 cancers in 185 countries. *CA Cancer J. Clin.* 71 209–249. 10.3322/caac.21660 33538338

[B19] Wang C. Yang Y. Zhang G. Li J. Wu X. Ma X. (2019a). Long noncoding RNA EMS connects c-Myc to cell cycle control and tumorigenesis. *Proc. Natl. Acad. Sci. U. S. A.* 116 14620–14629. 10.1073/pnas.1903432116 31262817 PMC6642410

[B20] Wang J. Xing H. Nikzad A. Liu B. Zhang Y. Li S. (2020). Long noncoding RNA MNX1 antisense RNA 1 exerts oncogenic functions in bladder cancer by regulating miR-218-5p/RAB1A axis. *J. Pharmacol. Exp. Ther.* 372 237–247. 10.1124/jpet.119.262949 31843814

[B21] Wang Y. Lu J. Wu Q. Jin Y. Wang D. Chen Y. (2019b). LncRNA LINRIS stabilizes IGF2BP2 and promotes the aerobic glycolysis in colorectal cancer. *Mol. Cancer* 18:174. 10.1186/s12943-019-1105-0 31791342 PMC6886219

[B22] Warburg O. (1956). On the origin of cancer cells. *Science* 123 309–314. 10.1126/science.123.3191.309 13298683

[B23] Weng M. Chen W. Chen X. Lu H. Sun Z. Yu Q. (2020). Fasting inhibits aerobic glycolysis and proliferation in colorectal cancer via the Fdft1-mediated AKT/mTOR/HIF1α pathway suppression. *Nat. Commun.* 11:1869. 10.1038/s41467-020-15795-8 32313017 PMC7170903

[B24] Yang F. Zhang H. Mei Y. Wu M. (2014). Reciprocal regulation of HIF-1α and lincRNA-p21 modulates the Warburg effect. *Mol. Cell* 53 88–100. 10.1016/j.molcel.2013.11.004 24316222

[B25] Zhang L. Fu Y. Guo H. (2019). c-Myc-induced long non-coding RNA small nucleolar RNA host gene 7 regulates glycolysis in breast cancer. *J. Breast Cancer* 22 533–547. 10.4048/jbc.2019.22.e54 31897328 PMC6933030

[B26] Zhang Z. Tan X. Luo J. Yao H. Si Z. Tong J. (2020). The miR-30a-5p/CLCF1 axis regulates sorafenib resistance and aerobic glycolysis in hepatocellular carcinoma. *Cell Death Dis.* 11:902. 10.1038/s41419-020-03123-3 33097691 PMC7584607

