# Supplementary material for: Identification of a Novel Glycolysis-Related LncRNA Signature for Predicting Overall Survival in Patients With Bladder Cancer
Source: Front Genet. 2021 Aug 19;12:720421. doi: 10.3389/fgene.2021.720421 (PMC8417422; doi:10.3389/fgene.2021.720421)
Supplement: Supplementary file 1 [file Table_1.DOCX]

| **Table S1.** Correlation between the prognostic glycolytic genes and lncRNAs in bladder cancer. | | | | |
| --- | --- | --- | --- | --- |
| Glycolytic gene | lncRNA | Correlation | pvalue | Regulation |
| CACNA1H | ZNF710-AS1 | 0.444633858 | 2.40E-21 | positive |
| SDC3 | ZNF710-AS1 | 0.405089082 | 1.16E-17 | positive |
| HDAC4 | ZNF710-AS1 | 0.466244046 | 1.43E-23 | positive |
| ALDH1B1 | ZNF710-AS1 | 0.38916778 | 2.60E-16 | positive |
| ADH1C | COLCA1 | 0.306515603 | 2.17E-10 | positive |
| KIF20A | AC099850.3 | 0.657234015 | 3.53E-52 | positive |
| CENPA | AC099850.3 | 0.552943527 | 2.76E-34 | positive |
| CDK1 | AC099850.3 | 0.602057619 | 6.83E-42 | positive |
| AURKA | AC099850.3 | 0.641835593 | 4.28E-49 | positive |
| HMMR | AC099850.3 | 0.655475001 | 8.11E-52 | positive |
| DEPDC1 | AC099850.3 | 0.737917108 | 7.65E-72 | positive |
| CXCR4 | AL357033.4 | 0.352391481 | 1.84E-13 | positive |
| ADH1B | AP003071.4 | 0.460604234 | 5.63E-23 | positive |
| CACNA1H | AP003071.4 | 0.696841417 | 5.18E-61 | positive |
| SDC3 | AP003071.4 | 0.330112467 | 6.63E-12 | positive |
| DCN | AP003071.4 | 0.749593704 | 2.65E-75 | positive |
| PRKAG2 | AP003071.4 | 0.350311238 | 2.61E-13 | positive |
| ALDH1B1 | AP003071.4 | 0.715093953 | 1.38E-65 | positive |
| TPST1 | AP003071.4 | 0.375994751 | 3.00E-15 | positive |
| SDC2 | AL589843.1 | 0.302626468 | 3.75E-10 | positive |
| ADH1B | AC005180.2 | 0.399418801 | 3.57E-17 | positive |
| CACNA1H | AC005180.2 | 0.814140571 | 1.35E-98 | positive |
| SDC3 | AC005180.2 | 0.348970768 | 3.25E-13 | positive |
| DCN | AC005180.2 | 0.424297422 | 2.16E-19 | positive |
| HDAC4 | AC005180.2 | 0.370840478 | 7.60E-15 | positive |
| PRKAG2 | AC005180.2 | 0.408095209 | 6.32E-18 | positive |
| ALDH1B1 | AC005180.2 | 0.805720861 | 4.54E-95 | positive |
| IER3 | IER3-AS1 | 0.609799592 | 3.25E-43 | positive |
| GPC3 | PWAR6 | 0.441176462 | 5.28E-21 | positive |
| CACNA1H | FENDRR | 0.530733315 | 3.06E-31 | positive |
| SDC3 | FENDRR | 0.369289131 | 1.00E-14 | positive |
| HDAC4 | FENDRR | 0.432428589 | 3.71E-20 | positive |
| ALDH1B1 | FENDRR | 0.430475281 | 5.69E-20 | positive |
| CACNA1H | DIO3OS | 0.328130268 | 8.99E-12 | positive |
| DCN | DIO3OS | 0.332932462 | 4.28E-12 | positive |
| ADH1B | MAGI2-AS3 | 0.340815377 | 1.23E-12 | positive |
| CACNA1H | MAGI2-AS3 | 0.415163894 | 1.48E-18 | positive |
| SDC3 | MAGI2-AS3 | 0.317471763 | 4.47E-11 | positive |
| DCN | MAGI2-AS3 | 0.498050306 | 3.76E-27 | positive |
| HDAC4 | MAGI2-AS3 | 0.369528797 | 9.60E-15 | positive |
| ALDH1B1 | MAGI2-AS3 | 0.441805872 | 4.57E-21 | positive |
| TPST1 | MAGI2-AS3 | 0.30778326 | 1.82E-10 | positive |
| KIF20A | LINC01082 | -0.301585087 | 4.34E-10 | positive |
| CACNA1H | LINC01082 | 0.480844809 | 3.61E-25 | positive |
| CDK1 | LINC01082 | -0.312321485 | 9.47E-11 | positive |
| AURKA | LINC01082 | -0.321661483 | 2.40E-11 | positive |
| DCN | LINC01082 | 0.332776851 | 4.38E-12 | positive |
| PRKACB | FIRRE | 0.321605284 | 2.42E-11 | positive |
| GPC3 | FIRRE | 0.379176417 | 1.68E-15 | positive |
| PRKAG2 | PRKAG2-AS1 | 0.319347598 | 3.38E-11 | positive |
| ADH1B | AC005180.1 | 0.374309517 | 4.08E-15 | positive |
| CACNA1H | AC005180.1 | 0.808431842 | 3.48E-96 | positive |
| SDC3 | AC005180.1 | 0.344019002 | 7.33E-13 | positive |
| DCN | AC005180.1 | 0.409023042 | 5.23E-18 | positive |
| HDAC4 | AC005180.1 | 0.369111899 | 1.03E-14 | positive |
| PRKAG2 | AC005180.1 | 0.400332447 | 2.99E-17 | positive |
| ALDH1B1 | AC005180.1 | 0.798066375 | 5.20E-92 | positive |
| PRKACB | AC024075.1 | 0.305121254 | 2.65E-10 | positive |
| HDAC4 | AC024075.1 | 0.348917876 | 3.28E-13 | positive |
| KIF20A | SNHG1 | 0.307912013 | 1.78E-10 | positive |
| CDK1 | SNHG1 | 0.308376875 | 1.67E-10 | positive |
| SPAG4 | MIR200CHG | 0.333187248 | 4.11E-12 | positive |
| AURKA | MIR200CHG | -0.312511753 | 9.22E-11 | positive |
| HMMR | MIR200CHG | -0.326231292 | 1.20E-11 | positive |
| SDC1 | MIR200CHG | 0.300035949 | 5.38E-10 | positive |
| DEPDC1 | MIR200CHG | -0.305711782 | 2.43E-10 | positive |
| TPST1 | NR2F1-AS1 | 0.305266164 | 2.59E-10 | positive |
| CACNA1H | AP001107.5 | 0.558558125 | 4.31E-35 | positive |
| SDC3 | AP001107.5 | 0.306811145 | 2.08E-10 | positive |
| DCN | AP001107.5 | 0.348234562 | 3.68E-13 | positive |
| ALDH1B1 | AP001107.5 | 0.540666947 | 1.42E-32 | positive |
| SPAG4 | AC016773.1 | 0.325246719 | 1.40E-11 | positive |
| CENPA | AC104794.2 | 0.374116308 | 4.22E-15 | positive |
| NUP210 | AC104794.2 | 0.346401003 | 4.97E-13 | positive |
| STMN1 | AC104794.2 | 0.470171902 | 5.40E-24 | positive |
| ME1 | AP002884.1 | 0.40244898 | 1.96E-17 | positive |
| HDAC4 | AP002884.1 | 0.39133396 | 1.72E-16 | positive |
| STMN1 | AP000251.1 | 0.338482186 | 1.78E-12 | positive |
| B3GNT3 | SPINT1-AS1 | 0.378588009 | 1.87E-15 | positive |
| SPAG4 | SPINT1-AS1 | 0.414384609 | 1.74E-18 | positive |
| SDC1 | SPINT1-AS1 | 0.365351502 | 2.00E-14 | positive |
| PAM | SPINT1-AS1 | -0.305028399 | 2.68E-10 | positive |
| SDC3 | AL049555.1 | -0.325396681 | 1.36E-11 | positive |
| GPC3 | AC018521.6 | 0.503503249 | 8.38E-28 | positive |
| SDC3 | AC105942.1 | 0.302845587 | 3.64E-10 | positive |
| DCN | AC105942.1 | 0.303536149 | 3.31E-10 | positive |
| ALDH1B1 | AC105942.1 | 0.358042163 | 7.09E-14 | positive |
| CACNA1H | AP003486.1 | 0.319482367 | 3.32E-11 | positive |
| HDAC4 | AP003486.1 | 0.311565436 | 1.06E-10 | positive |
| AURKA | AC104825.1 | -0.302933559 | 3.60E-10 | positive |
| KIF20A | MIR22HG | -0.336602353 | 2.40E-12 | positive |
| CENPA | MIR22HG | -0.400861923 | 2.69E-17 | positive |
| CDK1 | MIR22HG | -0.374492212 | 3.94E-15 | positive |
| AURKA | MIR22HG | -0.350443147 | 2.55E-13 | positive |
| DEPDC1 | MIR22HG | -0.358268736 | 6.82E-14 | positive |
| STMN1 | MIR22HG | -0.327833968 | 9.41E-12 | positive |
| DCN | AL049838.1 | 0.39760401 | 5.10E-17 | positive |
| CENPA | AL021807.1 | 0.364596242 | 2.29E-14 | positive |
| KIF20A | AC078778.1 | 0.313228816 | 8.31E-11 | positive |
| CENPA | AC078778.1 | 0.565599897 | 4.00E-36 | positive |
| CDK1 | AC078778.1 | 0.317882461 | 4.20E-11 | positive |
| AURKA | AC078778.1 | 0.326577738 | 1.14E-11 | positive |
| DEPDC1 | AC078778.1 | 0.357679574 | 7.55E-14 | positive |
| STMN1 | AC078778.1 | 0.349343565 | 3.06E-13 | positive |
| ADH1B | AL162424.1 | 0.332607491 | 4.50E-12 | positive |
| CACNA1H | AL162424.1 | 0.543359097 | 6.06E-33 | positive |
| DCN | AL162424.1 | 0.349938929 | 2.77E-13 | positive |
| HDAC4 | AL162424.1 | 0.357143297 | 8.27E-14 | positive |
| ALDH1B1 | AL162424.1 | 0.487079844 | 7.12E-26 | positive |
| GPC3 | SNHG14 | 0.387992385 | 3.24E-16 | positive |
| HDAC4 | AL137003.2 | 0.393711829 | 1.09E-16 | positive |
| ADH1B | MBNL1-AS1 | 0.361611027 | 3.84E-14 | positive |
| CACNA1H | MBNL1-AS1 | 0.729121361 | 2.34E-69 | positive |
| SDC3 | MBNL1-AS1 | 0.34935689 | 3.05E-13 | positive |
| DCN | MBNL1-AS1 | 0.430192126 | 6.06E-20 | positive |
| HDAC4 | MBNL1-AS1 | 0.375064913 | 3.55E-15 | positive |
| PRKAG2 | MBNL1-AS1 | 0.384931807 | 5.77E-16 | positive |
| ALDH1B1 | MBNL1-AS1 | 0.811614451 | 1.61E-97 | positive |
| ADH1B | AL136084.3 | 0.31960617 | 3.26E-11 | positive |
| CACNA1H | AL136084.3 | 0.42075472 | 4.60E-19 | positive |
| SDC3 | AL136084.3 | 0.337202822 | 2.19E-12 | positive |
| DCN | AL136084.3 | 0.440858314 | 5.67E-21 | positive |
| HDAC4 | AL136084.3 | 0.321256974 | 2.55E-11 | positive |
| ALDH1B1 | AL136084.3 | 0.454696352 | 2.31E-22 | positive |
| TPST1 | AL136084.3 | 0.339057444 | 1.63E-12 | positive |
| GPC3 | AC124312.5 | 0.383037707 | 8.22E-16 | positive |
| STMN1 | ZNF667-AS1 | -0.58914792 | 9.13E-40 | positive |
| ADH1B | AC053503.4 | 0.349859304 | 2.81E-13 | positive |
| CACNA1H | AC053503.4 | 0.814731051 | 7.53E-99 | positive |
| SDC3 | AC053503.4 | 0.315962717 | 5.58E-11 | positive |
| DCN | AC053503.4 | 0.402313184 | 2.02E-17 | positive |
| HDAC4 | AC053503.4 | 0.36916359 | 1.02E-14 | positive |
| PRKAG2 | AC053503.4 | 0.385933718 | 4.78E-16 | positive |
| ALDH1B1 | AC053503.4 | 0.778173314 | 1.21E-84 | positive |
| ADH1B | ACTA2-AS1 | 0.381239723 | 1.15E-15 | positive |
| CACNA1H | ACTA2-AS1 | 0.761125146 | 6.51E-79 | positive |
| SDC3 | ACTA2-AS1 | 0.341205386 | 1.15E-12 | positive |
| DCN | ACTA2-AS1 | 0.488899196 | 4.40E-26 | positive |
| HDAC4 | ACTA2-AS1 | 0.406288045 | 9.10E-18 | positive |
| PRKAG2 | ACTA2-AS1 | 0.360614099 | 4.56E-14 | positive |
| ALDH1B1 | ACTA2-AS1 | 0.692850922 | 4.67E-60 | positive |
| TPST1 | ACTA2-AS1 | 0.323434135 | 1.84E-11 | positive |
| IER3 | AC020916.1 | 0.322443084 | 2.13E-11 | positive |
| CENPA | DCST1-AS1 | 0.300855654 | 4.80E-10 | positive |
| HDAC4 | LINC00641 | 0.321897958 | 2.31E-11 | positive |
| DCN | LINC01614 | 0.399189078 | 3.74E-17 | positive |
| SDC2 | LINC01614 | 0.42151239 | 3.92E-19 | positive |
| PRKACB | AL353804.1 | 0.337841576 | 1.98E-12 | positive |
| HDAC4 | AL353804.1 | 0.311566462 | 1.06E-10 | positive |
| SPAG4 | MNX1-AS1 | 0.344676105 | 6.58E-13 | positive |
| ADH1B | AL583785.1 | 0.567808627 | 1.87E-36 | positive |
| DCN | AL583785.1 | 0.595648381 | 7.98E-41 | positive |
| GPC3 | AC011503.2 | 0.932382873 | 7.56E-183 | positive |
| ADH1B | MIR100HG | 0.419357258 | 6.17E-19 | positive |
| CACNA1H | MIR100HG | 0.594763845 | 1.12E-40 | positive |
| SDC3 | MIR100HG | 0.435580234 | 1.85E-20 | positive |
| DCN | MIR100HG | 0.621115157 | 3.26E-45 | positive |
| HDAC4 | MIR100HG | 0.439295507 | 8.06E-21 | positive |
| ALDH1B1 | MIR100HG | 0.629843933 | 8.20E-47 | positive |
| TPST1 | MIR100HG | 0.391916021 | 1.54E-16 | positive |
| CACNA1H | MAFG.DT | 0.35609511 | 9.88E-14 | positive |
| DCN | MAFG.DT | 0.319869171 | 3.13E-11 | positive |
| ALDH1B1 | MAFG.DT | 0.465517469 | 1.70E-23 | positive |
| DCN | BX322562.1 | 0.404228074 | 1.38E-17 | positive |
| ALDH1B1 | BX322562.1 | 0.308771956 | 1.58E-10 | positive |
| TPST1 | BX322562.1 | 0.315931289 | 5.60E-11 | positive |
| CACNA1H | AL122035.1 | 0.321105925 | 2.60E-11 | positive |
| PAM | AL122035.1 | 0.300225065 | 5.24E-10 | positive |
| HDAC4 | AL122035.1 | 0.337082911 | 2.23E-12 | positive |
| CXCR4 | AL117336.2 | 0.316683461 | 5.02E-11 | positive |
| SPAG4 | PCAT6 | 0.371980491 | 6.20E-15 | positive |
| ADH1B | PGM5-AS1 | 0.381561594 | 1.08E-15 | positive |
| CACNA1H | PGM5-AS1 | 0.523260826 | 2.88E-30 | positive |
| ALDH1B1 | PGM5-AS1 | 0.409554162 | 4.70E-18 | positive |
