# Supplementary material for: Identification of a Novel Glycolysis-Related LncRNA Signature for Predicting Overall Survival in Patients With Bladder Cancer
Source: Front Genet. 2021 Aug 19;12:720421. doi: 10.3389/fgene.2021.720421 (PMC8417422; doi:10.3389/fgene.2021.720421)
Supplement: Supplementary file 2 [file Table_2.DOCX]

| **Table S2.** Clinical parameters and pathological stages of patients with bladder cancer in this research | | | | | | | | | |
| --- | --- | --- | --- | --- | --- | --- | --- | --- | --- |
| ID | futime | fustat | age | geder | grade | stage | T | N | M |
| TCGA-B4-5834 | 38 | 0 | 59 | MALE | G1 | Stage I | T1 | N0 | M0 |
| TCGA-B4-5378 | 175 | 0 | 62 | MALE | G2 | Stage I | T1 | N0 | M0 |
| TCGA-BP-4353 | 375 | 1 | 61 | MALE | G2 | Stage I | T1 | N0 | M0 |
| TCGA-A3-3380 | 567 | 0 | 54 | MALE | G2 | Stage I | T1 | N0 | M0 |
| TCGA-A3-3331 | 1257 | 0 | 86 | FEMALE | G2 | Stage I | T1 | N0 | M0 |
| TCGA-CZ-4859 | 1787 | 0 | 59 | FEMALE | G2 | Stage I | T1 | N0 | M0 |
| TCGA-B0-5699 | 2741 | 0 | 53 | MALE | G2 | Stage I | T1 | N0 | M0 |
| TCGA-AK-3425 | 3343 | 0 | 68 | MALE | G2 | Stage I | T1 | N0 | M0 |
| TCGA-B0-5705 | 3668 | 0 | 65 | FEMALE | G2 | Stage I | T1 | N0 | M0 |
| TCGA-CZ-5986 | 373 | 0 | 61 | MALE | G3 | Stage I | T1 | N0 | M0 |
| TCGA-A3-3378 | 630 | 0 | 60 | MALE | G3 | Stage I | T1 | N0 | M0 |
| TCGA-B8-4154 | 255 | 0 | 73 | FEMALE | G2 | Stage I | T1 | N0 | M0 |
| TCGA-3Z-A93Z | 385 | 0 | 69 | MALE | G2 | Stage I | T1 | N0 | M0 |
| TCGA-BP-4768 | 400 | 0 | 72 | FEMALE | G2 | Stage I | T1 | N0 | M0 |
| TCGA-B0-4823 | 454 | 1 | 88 | MALE | G2 | Stage I | T1 | N0 | M0 |
| TCGA-B0-5120 | 493 | 0 | 72 | FEMALE | G2 | Stage I | T1 | N0 | M0 |
| TCGA-B8-5545 | 522 | 0 | 42 | MALE | G2 | Stage I | T1 | N0 | M0 |
| TCGA-A3-3387 | 617 | 0 | 49 | MALE | G2 | Stage I | T1 | N0 | M0 |
| TCGA-BP-4795 | 620 | 0 | 74 | FEMALE | G2 | Stage I | T1 | N0 | M0 |
| TCGA-BP-5186 | 693 | 0 | 50 | FEMALE | G2 | Stage I | T1 | N0 | M0 |
| TCGA-BP-4988 | 828 | 1 | 72 | MALE | G2 | Stage I | T1 | N0 | M0 |
| TCGA-BP-5006 | 840 | 0 | 61 | MALE | G2 | Stage I | T1 | N0 | M0 |
| TCGA-B0-5700 | 1082 | 0 | 77 | MALE | G2 | Stage I | T1 | N0 | M0 |
| TCGA-B0-5110 | 1092 | 0 | 71 | FEMALE | G2 | Stage I | T1 | N0 | M0 |
| TCGA-A3-3358 | 1307 | 0 | 57 | FEMALE | G2 | Stage I | T1 | N0 | M0 |
| TCGA-AK-3450 | 1508 | 0 | 85 | FEMALE | G2 | Stage I | T1 | N0 | M0 |
| TCGA-A3-3362 | 1559 | 0 | 60 | FEMALE | G2 | Stage I | T1 | N0 | M0 |
| TCGA-B0-5106 | 1598 | 1 | 64 | MALE | G2 | Stage I | T1 | N0 | M0 |
| TCGA-A3-3376 | 1696 | 1 | 51 | MALE | G2 | Stage I | T1 | N0 | M0 |
| TCGA-B0-5697 | 1835 | 0 | 50 | MALE | G2 | Stage I | T1 | N0 | M0 |
| TCGA-BP-4964 | 1862 | 0 | 54 | FEMALE | G2 | Stage I | T1 | N0 | M0 |
| TCGA-A3-3385 | 1993 | 0 | 46 | FEMALE | G2 | Stage I | T1 | N0 | M0 |
| TCGA-B0-4945 | 2145 | 1 | 75 | FEMALE | G2 | Stage I | T1 | N0 | M0 |
| TCGA-BP-4331 | 2454 | 1 | 52 | MALE | G2 | Stage I | T1 | N0 | M0 |
| TCGA-A3-3359 | 2504 | 0 | 82 | FEMALE | G2 | Stage I | T1 | N0 | M0 |
| TCGA-B8-5159 | 240 | 0 | 61 | FEMALE | G3 | Stage I | T1 | N0 | M0 |
| TCGA-B2-5641 | 324 | 0 | 79 | MALE | G3 | Stage I | T1 | N0 | M0 |
| TCGA-B8-4148 | 379 | 0 | 63 | FEMALE | G3 | Stage I | T1 | N0 | M0 |
| TCGA-BP-4986 | 785 | 0 | 75 | MALE | G3 | Stage I | T1 | N0 | M0 |
| TCGA-B0-5083 | 1045 | 1 | 63 | MALE | G3 | Stage I | T1 | N0 | M0 |
| TCGA-BP-5182 | 1165 | 0 | 56 | MALE | G3 | Stage I | T1 | N0 | M0 |
| TCGA-B0-5077 | 1317 | 1 | 77 | MALE | G3 | Stage I | T1 | N0 | M0 |
| TCGA-B0-4824 | 1657 | 1 | 49 | FEMALE | G3 | Stage I | T1 | N0 | M0 |
| TCGA-B0-4834 | 2090 | 1 | 49 | MALE | G3 | Stage I | T1 | N0 | M0 |
| TCGA-CJ-6030 | 2299 | 1 | 65 | MALE | G3 | Stage I | T1 | N0 | M0 |
| TCGA-B0-5707 | 2828 | 0 | 39 | FEMALE | G3 | Stage I | T1 | N0 | M0 |
| TCGA-B0-5691 | 3431 | 0 | 66 | FEMALE | G3 | Stage I | T1 | N0 | M0 |
| TCGA-BP-4165 | 3037 | 0 | 64 | FEMALE | G1 | Stage I | T1 | N0 | M0 |
| TCGA-B0-5119 | 59 | 0 | 61 | FEMALE | G2 | Stage I | T1 | N0 | M0 |
| TCGA-B4-5836 | 141 | 0 | 61 | FEMALE | G2 | Stage I | T1 | N0 | M0 |
| TCGA-B2-5633 | 358 | 0 | 56 | MALE | G2 | Stage I | T1 | N0 | M0 |
| TCGA-BP-4756 | 374 | 0 | 62 | FEMALE | G2 | Stage I | T1 | N0 | M0 |
| TCGA-B8-5553 | 435 | 0 | 67 | FEMALE | G2 | Stage I | T1 | N0 | M0 |
| TCGA-B8-5546 | 505 | 0 | 38 | FEMALE | G2 | Stage I | T1 | N0 | M0 |
| TCGA-B0-5121 | 554 | 0 | 56 | MALE | G2 | Stage I | T1 | N0 | M0 |
| TCGA-BP-4340 | 562 | 1 | 70 | FEMALE | G2 | Stage I | T1 | N0 | M0 |
| TCGA-B0-5399 | 652 | 0 | 46 | MALE | G2 | Stage I | T1 | N0 | M0 |
| TCGA-CZ-5988 | 693 | 0 | 38 | MALE | G2 | Stage I | T1 | N0 | M0 |
| TCGA-A3-3374 | 1314 | 0 | 51 | FEMALE | G2 | Stage I | T1 | N0 | M0 |
| TCGA-A3-3328 | 1385 | 0 | 79 | MALE | G2 | Stage I | T1 | N0 | M0 |
| TCGA-A3-3349 | 1385 | 0 | 34 | FEMALE | G2 | Stage I | T1 | N0 | M0 |
| TCGA-CZ-4854 | 1404 | 1 | 68 | MALE | G2 | Stage I | T1 | N0 | M0 |
| TCGA-B0-5695 | 1420 | 0 | 61 | FEMALE | G2 | Stage I | T1 | N0 | M0 |
| TCGA-B0-5710 | 1459 | 0 | 57 | MALE | G2 | Stage I | T1 | N0 | M0 |
| TCGA-CJ-4892 | 1521 | 0 | 65 | FEMALE | G2 | Stage I | T1 | N0 | M0 |
| TCGA-B0-5702 | 1605 | 0 | 71 | MALE | G2 | Stage I | T1 | N0 | M0 |
| TCGA-A3-3329 | 1624 | 0 | 75 | MALE | G2 | Stage I | T1 | N0 | M0 |
| TCGA-BP-4326 | 1625 | 1 | 53 | FEMALE | G2 | Stage I | T1 | N0 | M0 |
| TCGA-B0-4839 | 1639 | 1 | 80 | FEMALE | G2 | Stage I | T1 | N0 | M0 |
| TCGA-A3-3370 | 2274 | 0 | 48 | FEMALE | G2 | Stage I | T1 | N0 | M0 |
| TCGA-BP-4170 | 2343 | 1 | 72 | FEMALE | G2 | Stage I | T1 | N0 | M0 |
| TCGA-B0-4833 | 2386 | 1 | 82 | FEMALE | G2 | Stage I | T1 | N0 | M0 |
| TCGA-BP-4159 | 2601 | 1 | 70 | MALE | G2 | Stage I | T1 | N0 | M0 |
| TCGA-BP-4325 | 2964 | 0 | 64 | FEMALE | G2 | Stage I | T1 | N0 | M0 |
| TCGA-BP-4162 | 3074 | 0 | 65 | FEMALE | G2 | Stage I | T1 | N0 | M0 |
| TCGA-CW-6088 | 3222 | 0 | 60 | MALE | G2 | Stage I | T1 | N0 | M0 |
| TCGA-BP-4158 | 3377 | 0 | 69 | MALE | G2 | Stage I | T1 | N0 | M0 |
| TCGA-B8-5549 | 194 | 0 | 53 | MALE | G3 | Stage I | T1 | N0 | M0 |
| TCGA-B8-4621 | 431 | 0 | 63 | MALE | G3 | Stage I | T1 | N0 | M0 |
| TCGA-B0-5088 | 563 | 1 | 53 | MALE | G3 | Stage I | T1 | N0 | M0 |
| TCGA-A3-3313 | 735 | 1 | 59 | MALE | G3 | Stage I | T1 | N0 | M0 |
| TCGA-B0-4838 | 834 | 1 | 69 | FEMALE | G3 | Stage I | T1 | N0 | M0 |
| TCGA-A3-3306 | 1120 | 0 | 67 | MALE | G3 | Stage I | T1 | N0 | M0 |
| TCGA-B0-5703 | 1203 | 0 | 73 | MALE | G3 | Stage I | T1 | N0 | M0 |
| TCGA-BP-4995 | 1371 | 0 | 68 | MALE | G3 | Stage I | T1 | N0 | M0 |
| TCGA-B0-4837 | 1378 | 1 | 63 | MALE | G3 | Stage I | T1 | N0 | M0 |
| TCGA-CZ-5984 | 1491 | 0 | 51 | MALE | G3 | Stage I | T1 | N0 | M0 |
| TCGA-A3-3373 | 1621 | 0 | 54 | FEMALE | G3 | Stage I | T1 | N0 | M0 |
| TCGA-BP-4968 | 1746 | 0 | 40 | MALE | G3 | Stage I | T1 | N0 | M0 |
| TCGA-A3-3367 | 2270 | 0 | 72 | MALE | G3 | Stage I | T1 | N0 | M0 |
| TCGA-CJ-4874 | 2283 | 0 | 73 | FEMALE | G3 | Stage I | T1 | N0 | M0 |
| TCGA-B0-5698 | 2583 | 0 | 77 | MALE | G3 | Stage I | T1 | N0 | M0 |
| TCGA-BP-4338 | 2859 | 0 | 43 | MALE | G3 | Stage I | T1 | N0 | M0 |
| TCGA-BP-5169 | 193 | 0 | 70 | MALE | G4 | Stage I | T1 | N0 | M0 |
| TCGA-CJ-4872 | 1435 | 0 | 51 | MALE | G4 | Stage I | T1 | N0 | M0 |
| TCGA-G6-A8L7 | 2133 | 0 | 81 | FEMALE | G3 | Stage I | T1 | N0 | M0 |
| TCGA-EU-5904 | 551 | 0 | 47 | FEMALE | G1 | Stage I | T1 | N0 | M0 |
| TCGA-A3-3383 | 861 | 0 | 52 | MALE | G2 | Stage I | T1 | N0 | M0 |
| TCGA-A3-3311 | 1191 | 1 | 57 | MALE | G2 | Stage I | T1 | N0 | M0 |
| TCGA-EU-5905 | 119 | 0 | 67 | FEMALE | G3 | Stage I | T1 | N0 | M0 |
| TCGA-B0-5098 | 1584 | 1 | 53 | FEMALE | G3 | Stage I | T1 | N0 | M0 |
| TCGA-CZ-4866 | 1768 | 0 | 79 | FEMALE | G3 | Stage I | T1 | N0 | M0 |
| TCGA-B0-5102 | 2764 | 1 | 74 | FEMALE | G3 | Stage I | T1 | N0 | M0 |
| TCGA-A3-3336 | 1043 | 0 | 75 | FEMALE | G4 | Stage I | T1 | N0 | M0 |
| TCGA-AS-3778 | 43 | 0 | 35 | MALE | G1 | Stage I | T1 | N0 | M0 |
| TCGA-A3-3326 | 1137 | 0 | 47 | MALE | G1 | Stage I | T1 | N0 | M0 |
| TCGA-CW-6093 | 3146 | 0 | 73 | MALE | G1 | Stage I | T1 | N0 | M0 |
| TCGA-BP-5173 | 62 | 1 | 75 | MALE | G2 | Stage I | T1 | N0 | M0 |
| TCGA-CZ-4865 | 166 | 1 | 70 | FEMALE | G2 | Stage I | T1 | N0 | M0 |
| TCGA-B2-5636 | 265 | 0 | 79 | MALE | G2 | Stage I | T1 | N0 | M0 |
| TCGA-B2-5635 | 315 | 0 | 74 | MALE | G2 | Stage I | T1 | N0 | M0 |
| TCGA-BP-4782 | 354 | 0 | 55 | FEMALE | G2 | Stage I | T1 | N0 | M0 |
| TCGA-DV-5569 | 355 | 0 | 29 | FEMALE | G2 | Stage I | T1 | N0 | M0 |
| TCGA-DV-5568 | 370 | 0 | 26 | MALE | G2 | Stage I | T1 | N0 | M0 |
| TCGA-BP-4349 | 372 | 0 | 68 | FEMALE | G2 | Stage I | T1 | N0 | M0 |
| TCGA-BP-5187 | 406 | 0 | 54 | MALE | G2 | Stage I | T1 | N0 | M0 |
| TCGA-BP-5194 | 408 | 0 | 39 | MALE | G2 | Stage I | T1 | N0 | M0 |
| TCGA-BP-4776 | 411 | 0 | 52 | MALE | G2 | Stage I | T1 | N0 | M0 |
| TCGA-BP-5192 | 714 | 0 | 59 | MALE | G2 | Stage I | T1 | N0 | M0 |
| TCGA-DV-5574 | 723 | 0 | 37 | MALE | G2 | Stage I | T1 | N0 | M0 |
| TCGA-DV-5576 | 727 | 1 | 55 | FEMALE | G2 | Stage I | T1 | N0 | M0 |
| TCGA-BP-5195 | 749 | 0 | 75 | MALE | G2 | Stage I | T1 | N0 | M0 |
| TCGA-CZ-4853 | 774 | 0 | 82 | MALE | G2 | Stage I | T1 | N0 | M0 |
| TCGA-AK-3461 | 853 | 0 | 72 | MALE | G2 | Stage I | T1 | N0 | M0 |
| TCGA-A3-3365 | 873 | 0 | 46 | MALE | G2 | Stage I | T1 | N0 | M0 |
| TCGA-DV-5567 | 910 | 0 | 40 | FEMALE | G2 | Stage I | T1 | N0 | M0 |
| TCGA-AK-3460 | 951 | 0 | 58 | MALE | G2 | Stage I | T1 | N0 | M0 |
| TCGA-DV-5575 | 1006 | 0 | 52 | FEMALE | G2 | Stage I | T1 | N0 | M0 |
| TCGA-BP-5196 | 1018 | 0 | 53 | MALE | G2 | Stage I | T1 | N0 | M0 |
| TCGA-BP-5008 | 1071 | 0 | 46 | MALE | G2 | Stage I | T1 | N0 | M0 |
| TCGA-BP-4790 | 1111 | 1 | 76 | MALE | G2 | Stage I | T1 | N0 | M0 |
| TCGA-BP-4801 | 1124 | 0 | 57 | MALE | G2 | Stage I | T1 | N0 | M0 |
| TCGA-DV-5573 | 1130 | 0 | 41 | MALE | G2 | Stage I | T1 | N0 | M0 |
| TCGA-A3-3325 | 1170 | 1 | 52 | MALE | G2 | Stage I | T1 | N0 | M0 |
| TCGA-BP-4999 | 1266 | 0 | 56 | MALE | G2 | Stage I | T1 | N0 | M0 |
| TCGA-BP-4763 | 1270 | 1 | 79 | FEMALE | G2 | Stage I | T1 | N0 | M0 |
| TCGA-DV-5565 | 1329 | 0 | 59 | MALE | G2 | Stage I | T1 | N0 | M0 |
| TCGA-DV-5566 | 1398 | 0 | 67 | FEMALE | G2 | Stage I | T1 | N0 | M0 |
| TCGA-BP-4991 | 1413 | 0 | 54 | MALE | G2 | Stage I | T1 | N0 | M0 |
| TCGA-BP-5168 | 1463 | 1 | 75 | MALE | G2 | Stage I | T1 | N0 | M0 |
| TCGA-A3-3322 | 1478 | 0 | 51 | MALE | G2 | Stage I | T1 | N0 | M0 |
| TCGA-BP-4789 | 1489 | 0 | 48 | MALE | G2 | Stage I | T1 | N0 | M0 |
| TCGA-CJ-4905 | 1496 | 0 | 62 | FEMALE | G2 | Stage I | T1 | N0 | M0 |
| TCGA-CJ-4908 | 1531 | 0 | 38 | MALE | G2 | Stage I | T1 | N0 | M0 |
| TCGA-BP-5176 | 1590 | 1 | 78 | FEMALE | G2 | Stage I | T1 | N0 | M0 |
| TCGA-BP-4344 | 1666 | 0 | 75 | FEMALE | G2 | Stage I | T1 | N0 | M0 |
| TCGA-BP-4177 | 1670 | 0 | 65 | MALE | G2 | Stage I | T1 | N0 | M0 |
| TCGA-BP-4969 | 1794 | 0 | 63 | FEMALE | G2 | Stage I | T1 | N0 | M0 |
| TCGA-BP-4775 | 1843 | 0 | 55 | FEMALE | G2 | Stage I | T1 | N0 | M0 |
| TCGA-BP-4784 | 1854 | 0 | 67 | FEMALE | G2 | Stage I | T1 | N0 | M0 |
| TCGA-BP-4965 | 1871 | 0 | 46 | MALE | G2 | Stage I | T1 | N0 | M0 |
| TCGA-BP-4769 | 1876 | 0 | 63 | MALE | G2 | Stage I | T1 | N0 | M0 |
| TCGA-BP-4774 | 1885 | 0 | 57 | FEMALE | G2 | Stage I | T1 | N0 | M0 |
| TCGA-BP-4961 | 1935 | 0 | 47 | MALE | G2 | Stage I | T1 | N0 | M0 |
| TCGA-CW-5588 | 2017 | 0 | 78 | FEMALE | G2 | Stage I | T1 | N0 | M0 |
| TCGA-CZ-5982 | 2042 | 0 | 59 | FEMALE | G2 | Stage I | T1 | N0 | M0 |
| TCGA-BP-4765 | 2184 | 0 | 43 | MALE | G2 | Stage I | T1 | N0 | M0 |
| TCGA-BP-4758 | 2208 | 0 | 40 | MALE | G2 | Stage I | T1 | N0 | M0 |
| TCGA-BP-5174 | 2257 | 0 | 45 | FEMALE | G2 | Stage I | T1 | N0 | M0 |
| TCGA-BP-5180 | 2263 | 0 | 53 | MALE | G2 | Stage I | T1 | N0 | M0 |
| TCGA-BP-4760 | 2361 | 0 | 69 | MALE | G2 | Stage I | T1 | N0 | M0 |
| TCGA-BP-4759 | 2372 | 0 | 50 | MALE | G2 | Stage I | T1 | N0 | M0 |
| TCGA-CW-5589 | 2378 | 0 | 52 | MALE | G2 | Stage I | T1 | N0 | M0 |
| TCGA-BP-5170 | 2412 | 0 | 55 | MALE | G2 | Stage I | T1 | N0 | M0 |
| TCGA-CW-5583 | 2489 | 0 | 51 | FEMALE | G2 | Stage I | T1 | N0 | M0 |
| TCGA-CW-6096 | 2701 | 0 | 44 | FEMALE | G2 | Stage I | T1 | N0 | M0 |
| TCGA-BP-4993 | 177 | 0 | 58 | MALE | G3 | Stage I | T1 | N0 | M0 |
| TCGA-BP-4807 | 211 | 0 | 42 | MALE | G3 | Stage I | T1 | N0 | M0 |
| TCGA-BP-5177 | 293 | 0 | 46 | FEMALE | G3 | Stage I | T1 | N0 | M0 |
| TCGA-B2-4099 | 374 | 0 | 83 | MALE | G3 | Stage I | T1 | N0 | M0 |
| TCGA-BP-5175 | 932 | 0 | 60 | MALE | G3 | Stage I | T1 | N0 | M0 |
| TCGA-BP-4998 | 932 | 0 | 49 | MALE | G3 | Stage I | T1 | N0 | M0 |
| TCGA-BP-5190 | 1011 | 0 | 61 | MALE | G3 | Stage I | T1 | N0 | M0 |
| TCGA-BP-5004 | 1126 | 0 | 53 | MALE | G3 | Stage I | T1 | N0 | M0 |
| TCGA-BP-5185 | 1132 | 0 | 56 | MALE | G3 | Stage I | T1 | N0 | M0 |
| TCGA-BP-5184 | 1133 | 0 | 54 | MALE | G3 | Stage I | T1 | N0 | M0 |
| TCGA-BP-4994 | 1308 | 0 | 54 | MALE | G3 | Stage I | T1 | N0 | M0 |
| TCGA-BP-4762 | 1343 | 1 | 42 | MALE | G3 | Stage I | T1 | N0 | M0 |
| TCGA-BP-4766 | 1462 | 0 | 43 | FEMALE | G3 | Stage I | T1 | N0 | M0 |
| TCGA-BP-4976 | 1632 | 0 | 77 | MALE | G3 | Stage I | T1 | N0 | M0 |
| TCGA-BP-4777 | 1731 | 0 | 46 | MALE | G3 | Stage I | T1 | N0 | M0 |
| TCGA-AK-3440 | 1745 | 0 | 58 | MALE | G3 | Stage I | T1 | N0 | M0 |
| TCGA-CJ-5671 | 1943 | 0 | 51 | MALE | G3 | Stage I | T1 | N0 | M0 |
| TCGA-CJ-4886 | 1952 | 0 | 42 | FEMALE | G3 | Stage I | T1 | N0 | M0 |
| TCGA-CJ-5672 | 1972 | 1 | 84 | MALE | G3 | Stage I | T1 | N0 | M0 |
| TCGA-BP-4781 | 2080 | 0 | 78 | MALE | G3 | Stage I | T1 | N0 | M0 |
| TCGA-CJ-6027 | 1855 | 0 | 77 | MALE | G4 | Stage I | T1 | N0 | M0 |
| TCGA-CJ-4889 | 1946 | 0 | 63 | FEMALE | G4 | Stage I | T1 | N0 | M0 |
| TCGA-T7-A92I | 356 | 0 | 47 | FEMALE | G1 | Stage I | T1 | N0 | M0 |
| TCGA-A3-A6NJ | 468 | 0 | 57 | FEMALE | G1 | Stage I | T1 | N0 | M0 |
| TCGA-B8-A54K | 469 | 0 | 61 | MALE | G1 | Stage I | T1 | N0 | M0 |
| TCGA-A3-A8OV | 340 | 0 | 75 | MALE | G2 | Stage I | T1 | N0 | M0 |
| TCGA-DV-A4VZ | 365 | 0 | 53 | MALE | G2 | Stage I | T1 | N0 | M0 |
| TCGA-MW-A4EC | 498 | 0 | 72 | FEMALE | G2 | Stage I | T1 | N0 | M0 |
| TCGA-B8-A54F | 519 | 0 | 49 | FEMALE | G2 | Stage I | T1 | N0 | M0 |
| TCGA-MM-A84U | 700 | 0 | 58 | FEMALE | G2 | Stage I | T1 | N0 | M0 |
| TCGA-B8-A54G | 53 | 0 | 50 | MALE | G3 | Stage I | T1 | N0 | M0 |
| TCGA-A3-A6NI | 1018 | 0 | 47 | MALE | G3 | Stage I | T1 | N0 | M0 |
| TCGA-B8-A7U6 | 495 | 0 | 54 | FEMALE | G3 | Stage I | T1 | N0 | M0 |
| TCGA-A3-3323 | 1106 | 0 | 53 | MALE | G1 | Stage I | T1 | N0 | M0 |
| TCGA-A3-3320 | 1508 | 0 | 52 | FEMALE | G1 | Stage I | T1 | N0 | M0 |
| TCGA-B0-5690 | 2408 | 0 | 53 | FEMALE | G1 | Stage I | T1 | N0 | M0 |
| TCGA-B2-4098 | 51 | 1 | 72 | FEMALE | G2 | Stage I | T1 | N0 | M0 |
| TCGA-CJ-4920 | 139 | 1 | 64 | FEMALE | G2 | Stage I | T1 | N0 | M0 |
| TCGA-B2-4102 | 202 | 0 | 61 | MALE | G2 | Stage I | T1 | N0 | M0 |
| TCGA-EU-5906 | 206 | 0 | 55 | MALE | G2 | Stage I | T1 | N0 | M0 |
| TCGA-B2-3924 | 371 | 0 | 73 | MALE | G2 | Stage I | T1 | N0 | M0 |
| TCGA-B8-5552 | 392 | 0 | 41 | FEMALE | G2 | Stage I | T1 | N0 | M0 |
| TCGA-B8-4146 | 511 | 0 | 41 | FEMALE | G2 | Stage I | T1 | N0 | M0 |
| TCGA-B0-5117 | 535 | 0 | 40 | MALE | G2 | Stage I | T1 | N0 | M0 |
| TCGA-BP-4987 | 1124 | 0 | 41 | FEMALE | G2 | Stage I | T1 | N0 | M0 |
| TCGA-A3-3319 | 1130 | 0 | 70 | MALE | G2 | Stage I | T1 | N0 | M0 |
| TCGA-BP-5001 | 1177 | 0 | 43 | FEMALE | G2 | Stage I | T1 | N0 | M0 |
| TCGA-BP-4804 | 1459 | 0 | 59 | MALE | G2 | Stage I | T1 | N0 | M0 |
| TCGA-AK-3444 | 1471 | 0 | 80 | FEMALE | G2 | Stage I | T1 | N0 | M0 |
| TCGA-BP-5181 | 1495 | 0 | 58 | FEMALE | G2 | Stage I | T1 | N0 | M0 |
| TCGA-CJ-4899 | 1528 | 0 | 42 | MALE | G2 | Stage I | T1 | N0 | M0 |
| TCGA-CJ-4634 | 1820 | 0 | 60 | FEMALE | G2 | Stage I | T1 | N0 | M0 |
| TCGA-CZ-4862 | 1843 | 0 | 46 | MALE | G2 | Stage I | T1 | N0 | M0 |
| TCGA-BP-4176 | 1955 | 0 | 64 | MALE | G2 | Stage I | T1 | N0 | M0 |
| TCGA-AK-3434 | 2087 | 0 | 72 | MALE | G2 | Stage I | T1 | N0 | M0 |
| TCGA-B0-5693 | 3076 | 0 | 47 | FEMALE | G2 | Stage I | T1 | N0 | M0 |
| TCGA-A3-3346 | 137 | 1 | 68 | MALE | G3 | Stage I | T1 | N0 | M0 |
| TCGA-BP-4977 | 454 | 0 | 57 | MALE | G3 | Stage I | T1 | N0 | M0 |
| TCGA-BP-5000 | 563 | 0 | 40 | MALE | G3 | Stage I | T1 | N0 | M0 |
| TCGA-A3-3382 | 574 | 0 | 69 | MALE | G3 | Stage I | T1 | N0 | M0 |
| TCGA-CJ-4893 | 750 | 0 | 76 | FEMALE | G3 | Stage I | T1 | N0 | M0 |
| TCGA-AK-3454 | 874 | 0 | 84 | MALE | G3 | Stage I | T1 | N0 | M0 |
| TCGA-BP-4982 | 1014 | 0 | 42 | MALE | G3 | Stage I | T1 | N0 | M0 |
| TCGA-BP-5009 | 1092 | 1 | 52 | MALE | G3 | Stage I | T1 | N0 | M0 |
| TCGA-AK-3458 | 1168 | 0 | 48 | MALE | G3 | Stage I | T1 | N0 | M0 |
| TCGA-A3-3324 | 1186 | 0 | 51 | MALE | G3 | Stage I | T1 | N0 | M0 |
| TCGA-CJ-4635 | 1416 | 0 | 48 | MALE | G3 | Stage I | T1 | N0 | M0 |
| TCGA-BP-4975 | 1433 | 0 | 40 | MALE | G3 | Stage I | T1 | N0 | M0 |
| TCGA-CJ-4903 | 1560 | 0 | 50 | MALE | G3 | Stage I | T1 | N0 | M0 |
| TCGA-BP-4963 | 1834 | 0 | 63 | MALE | G3 | Stage I | T1 | N0 | M0 |
| TCGA-CJ-5683 | 1889 | 0 | 78 | MALE | G3 | Stage I | T1 | N0 | M0 |
| TCGA-CJ-6031 | 1906 | 0 | 54 | MALE | G3 | Stage I | T1 | N0 | M0 |
| TCGA-CJ-5686 | 2038 | 0 | 59 | FEMALE | G3 | Stage I | T1 | N0 | M0 |
| TCGA-CW-6090 | 2552 | 0 | 68 | MALE | G3 | Stage I | T1 | N0 | M0 |
| TCGA-BP-4959 | 2660 | 0 | 49 | MALE | G3 | Stage I | T1 | N0 | M0 |
| TCGA-BP-4161 | 2746 | 0 | 74 | MALE | G3 | Stage I | T1 | N0 | M0 |
| TCGA-CW-5581 | 2799 | 0 | 44 | MALE | G3 | Stage I | T1 | N0 | M0 |
| TCGA-B0-5812 | 2963 | 0 | 53 | MALE | G3 | Stage I | T1 | N0 | M0 |
| TCGA-BP-4992 | 501 | 0 | 66 | MALE | G4 | Stage I | T1 | N0 | M0 |
| TCGA-BP-5189 | 822 | 1 | 60 | MALE | G4 | Stage I | T1 | N0 | M0 |
| TCGA-6D-AA2E | 362 | 0 | 68 | FEMALE | G2 | Stage I | T1 | N0 | M0 |
| TCGA-A3-A6NL | 689 | 0 | 49 | FEMALE | G2 | Stage I | T1 | N0 | M0 |
| TCGA-B8-A54I | 150 | 0 | 48 | MALE | G3 | Stage I | T1 | N0 | M0 |
| TCGA-B8-A54E | 909 | 0 | 62 | FEMALE | G3 | Stage I | T1 | N0 | M0 |
| TCGA-G6-A8L8 | 1091 | 1 | 62 | FEMALE | G3 | Stage I | T1 | N0 | M0 |
| TCGA-DV-A4W0 | 2008 | 0 | 55 | MALE | G3 | Stage I | T1 | N0 | M0 |
| TCGA-B8-A8YJ | 431 | 0 | 60 | FEMALE | G2 | Stage I | T1 | N0 | M0 |
| TCGA-B0-5092 | 459 | 1 | 53 | FEMALE | G3 | Stage IV | T1 | N0 | M1 |
| TCGA-G6-A5PC | 242 | 1 | 54 | FEMALE | G4 | Stage IV | T1 | N0 | M1 |
| TCGA-BP-4327 | 109 | 1 | 75 | FEMALE | G2 | Stage II | T2 | N0 | M0 |
| TCGA-A3-3363 | 319 | 0 | 50 | MALE | G2 | Stage II | T2 | N0 | M0 |
| TCGA-BP-4169 | 701 | 1 | 76 | FEMALE | G2 | Stage II | T2 | N0 | M0 |
| TCGA-CZ-5469 | 946 | 1 | 41 | MALE | G2 | Stage II | T2 | N0 | M0 |
| TCGA-B0-4852 | 1121 | 1 | 78 | FEMALE | G2 | Stage II | T2 | N0 | M0 |
| TCGA-BP-5007 | 1140 | 0 | 45 | MALE | G2 | Stage II | T2 | N0 | M0 |
| TCGA-A3-3317 | 1491 | 0 | 67 | MALE | G2 | Stage II | T2 | N0 | M0 |
| TCGA-CZ-5452 | 1556 | 0 | 69 | MALE | G2 | Stage II | T2 | N0 | M0 |
| TCGA-CZ-5989 | 1599 | 0 | 60 | MALE | G2 | Stage II | T2 | N0 | M0 |
| TCGA-CZ-5985 | 1629 | 0 | 58 | MALE | G2 | Stage II | T2 | N0 | M0 |
| TCGA-AK-3429 | 2017 | 0 | 54 | FEMALE | G2 | Stage II | T2 | N0 | M0 |
| TCGA-CJ-4639 | 2308 | 0 | 49 | FEMALE | G2 | Stage II | T2 | N0 | M0 |
| TCGA-B0-5706 | 2414 | 0 | 45 | MALE | G2 | Stage II | T2 | N0 | M0 |
| TCGA-A3-3343 | 945 | 0 | 79 | MALE | G3 | Stage II | T2 | N0 | M0 |
| TCGA-AK-3456 | 1143 | 0 | 48 | MALE | G3 | Stage II | T2 | N0 | M0 |
| TCGA-CZ-4864 | 1315 | 1 | 86 | MALE | G3 | Stage II | T2 | N0 | M0 |
| TCGA-B0-4816 | 1371 | 1 | 49 | MALE | G3 | Stage II | T2 | N0 | M0 |
| TCGA-AK-3451 | 1481 | 0 | 48 | MALE | G3 | Stage II | T2 | N0 | M0 |
| TCGA-CZ-5456 | 1558 | 0 | 57 | MALE | G3 | Stage II | T2 | N0 | M0 |
| TCGA-CZ-5451 | 1668 | 0 | 74 | MALE | G3 | Stage II | T2 | N0 | M0 |
| TCGA-BP-4174 | 1879 | 0 | 49 | MALE | G3 | Stage II | T2 | N0 | M0 |
| TCGA-BP-4173 | 1893 | 0 | 47 | MALE | G3 | Stage II | T2 | N0 | M0 |
| TCGA-BP-4960 | 2172 | 0 | 46 | MALE | G3 | Stage II | T2 | N0 | M0 |
| TCGA-BP-4342 | 2256 | 1 | 79 | MALE | G3 | Stage II | T2 | N0 | M0 |
| TCGA-A3-3357 | 2688 | 0 | 62 | MALE | G3 | Stage II | T2 | N0 | M0 |
| TCGA-BP-5199 | 1355 | 0 | 58 | MALE | G4 | Stage II | T2 | N0 | M0 |
| TCGA-A3-3351 | 910 | 0 | 42 | MALE | G2 | Stage II | T2 | N0 | M0 |
| TCGA-A3-3335 | 1886 | 0 | 41 | MALE | G4 | Stage II | T2 | N0 | M0 |
| TCGA-B8-A54H | 256 | 0 | 69 | FEMALE | G3 | Stage II | T2 | N0 | M0 |
| TCGA-CJ-4643 | 1793 | 0 | 67 | FEMALE | G3 | Stage II | T2 | N0 | M0 |
| TCGA-CJ-4876 | 1955 | 0 | 57 | MALE | G3 | Stage II | T2 | N0 | M0 |
| TCGA-B2-3923 | 362 | 0 | 59 | MALE | G2 | Stage II | T2 | N0 | M0 |
| TCGA-CZ-4861 | 446 | 1 | 63 | MALE | G2 | Stage II | T2 | N0 | M0 |
| TCGA-CZ-5463 | 662 | 0 | 76 | MALE | G2 | Stage II | T2 | N0 | M0 |
| TCGA-AK-3447 | 1217 | 0 | 83 | MALE | G2 | Stage II | T2 | N0 | M0 |
| TCGA-AK-3453 | 1397 | 0 | 58 | FEMALE | G2 | Stage II | T2 | N0 | M0 |
| TCGA-CJ-4642 | 1628 | 0 | 47 | MALE | G2 | Stage II | T2 | N0 | M0 |
| TCGA-BP-4962 | 1785 | 0 | 58 | MALE | G2 | Stage II | T2 | N0 | M0 |
| TCGA-B0-4818 | 510 | 1 | 68 | FEMALE | G3 | Stage II | T2 | N0 | M0 |
| TCGA-A3-3316 | 1493 | 0 | 57 | MALE | G3 | Stage II | T2 | N0 | M0 |
| TCGA-CJ-4912 | 1657 | 0 | 61 | MALE | G3 | Stage II | T2 | N0 | M0 |
| TCGA-AK-3431 | 1853 | 0 | 62 | FEMALE | G3 | Stage II | T2 | N0 | M0 |
| TCGA-CJ-6032 | 2548 | 0 | 63 | FEMALE | G3 | Stage II | T2 | N0 | M0 |
| TCGA-BP-5200 | 1063 | 0 | 44 | MALE | G4 | Stage II | T2 | N0 | M0 |
| TCGA-B0-4822 | 1111 | 1 | 78 | MALE | G4 | Stage II | T2 | N0 | M0 |
| TCGA-CZ-4858 | 1943 | 0 | 39 | MALE | G4 | Stage II | T2 | N0 | M0 |
| TCGA-B2-4101 | 188 | 0 | 52 | MALE | G3 | Stage II | T2 | N0 | M0 |
| TCGA-CJ-5675 | 2430 | 0 | 70 | MALE | G3 | Stage II | T2 | N0 | M0 |
| TCGA-B8-A54J | 528 | 0 | 60 | MALE | G2 | Stage II | T2 | N0 | M0 |
| TCGA-MM-A564 | 607 | 0 | 68 | MALE | G2 | Stage II | T2 | N0 | M0 |
| TCGA-CZ-5454 | 722 | 1 | 63 | MALE | G2 | Stage IV | T2 | N0 | M1 |
| TCGA-AK-3436 | 2044 | 0 | 40 | MALE | G2 | Stage IV | T2 | N0 | M1 |
| TCGA-B0-4691 | 139 | 1 | 55 | MALE | G3 | Stage IV | T2 | N0 | M1 |
| TCGA-B0-5115 | 797 | 0 | 43 | MALE | G3 | Stage IV | T2 | N0 | M1 |
| TCGA-B0-5712 | 2722 | 0 | 68 | FEMALE | G3 | Stage IV | T2 | N0 | M1 |
| TCGA-B0-5107 | 927 | 1 | 65 | FEMALE | G4 | Stage IV | T2 | N0 | M1 |
| TCGA-CJ-5678 | 574 | 1 | 62 | MALE | G3 | Stage IV | T2 | N0 | M1 |
| TCGA-BP-4967 | 205 | 0 | 76 | MALE | G2 | Stage III | T3 | N0 | M0 |
| TCGA-B8-4620 | 226 | 0 | 70 | FEMALE | G2 | Stage III | T3 | N0 | M0 |
| TCGA-B8-4151 | 280 | 0 | 51 | FEMALE | G2 | Stage III | T3 | N0 | M0 |
| TCGA-B0-5113 | 359 | 0 | 69 | FEMALE | G2 | Stage III | T3 | N0 | M0 |
| TCGA-B0-5075 | 637 | 1 | 77 | FEMALE | G2 | Stage III | T3 | N0 | M0 |
| TCGA-BP-4329 | 845 | 1 | 75 | MALE | G2 | Stage III | T3 | N0 | M0 |
| TCGA-B0-5108 | 911 | 0 | 54 | MALE | G2 | Stage III | T3 | N0 | M0 |
| TCGA-BP-5191 | 967 | 0 | 79 | MALE | G2 | Stage III | T3 | N0 | M0 |
| TCGA-BP-4351 | 970 | 0 | 51 | FEMALE | G2 | Stage III | T3 | N0 | M0 |
| TCGA-BP-4332 | 1133 | 0 | 36 | MALE | G2 | Stage III | T3 | N0 | M0 |
| TCGA-BP-4330 | 1888 | 0 | 60 | FEMALE | G2 | Stage III | T3 | N0 | M0 |
| TCGA-BP-4160 | 2881 | 0 | 67 | MALE | G2 | Stage III | T3 | N0 | M0 |
| TCGA-B0-4710 | 96 | 0 | 75 | FEMALE | G3 | Stage III | T3 | N0 | M0 |
| TCGA-BP-4989 | 118 | 0 | 58 | MALE | G3 | Stage III | T3 | N0 | M0 |
| TCGA-B0-5095 | 245 | 1 | 81 | MALE | G3 | Stage III | T3 | N0 | M0 |
| TCGA-B0-4843 | 320 | 1 | 57 | MALE | G3 | Stage III | T3 | N0 | M0 |
| TCGA-B8-5550 | 434 | 0 | 71 | MALE | G3 | Stage III | T3 | N0 | M0 |
| TCGA-B0-5694 | 480 | 1 | 71 | MALE | G3 | Stage III | T3 | N0 | M0 |
| TCGA-A3-3352 | 561 | 1 | 74 | MALE | G3 | Stage III | T3 | N0 | M0 |
| TCGA-BP-4334 | 645 | 1 | 56 | MALE | G3 | Stage III | T3 | N0 | M0 |
| TCGA-B0-5085 | 770 | 1 | 76 | FEMALE | G3 | Stage III | T3 | N0 | M0 |
| TCGA-CJ-4894 | 841 | 1 | 58 | MALE | G3 | Stage III | T3 | N0 | M0 |
| TCGA-B0-4696 | 866 | 1 | 58 | MALE | G3 | Stage III | T3 | N0 | M0 |
| TCGA-B0-4811 | 1417 | 1 | 48 | MALE | G3 | Stage III | T3 | N0 | M0 |
| TCGA-BP-4971 | 1487 | 0 | 40 | MALE | G3 | Stage III | T3 | N0 | M0 |
| TCGA-CJ-4873 | 1776 | 0 | 85 | FEMALE | G3 | Stage III | T3 | N0 | M0 |
| TCGA-BP-4343 | 1912 | 1 | 64 | MALE | G3 | Stage III | T3 | N0 | M0 |
| TCGA-CJ-4636 | 1924 | 0 | 51 | MALE | G3 | Stage III | T3 | N0 | M0 |
| TCGA-BP-4163 | 2839 | 0 | 60 | FEMALE | G3 | Stage III | T3 | N0 | M0 |
| TCGA-CZ-5467 | 73 | 1 | 86 | FEMALE | G4 | Stage III | T3 | N0 | M0 |
| TCGA-B0-4693 | 77 | 1 | 72 | FEMALE | G4 | Stage III | T3 | N0 | M0 |
| TCGA-BP-5010 | 878 | 1 | 63 | MALE | G4 | Stage III | T3 | N0 | M0 |
| TCGA-BP-4985 | 952 | 1 | 72 | MALE | G4 | Stage III | T3 | N0 | M0 |
| TCGA-B0-4842 | 1724 | 1 | 73 | FEMALE | G4 | Stage III | T3 | N0 | M0 |
| TCGA-B0-5696 | 1727 | 0 | 69 | MALE | G4 | Stage III | T3 | N0 | M0 |
| TCGA-CJ-4640 | 1998 | 0 | 49 | MALE | G4 | Stage III | T3 | N0 | M0 |
| TCGA-B4-5832 | 155 | 0 | 65 | MALE | G2 | Stage III | T3 | N0 | M0 |
| TCGA-B0-5081 | 362 | 1 | 79 | FEMALE | G2 | Stage III | T3 | N0 | M0 |
| TCGA-B0-5097 | 665 | 0 | 59 | FEMALE | G2 | Stage III | T3 | N0 | M0 |
| TCGA-AK-3428 | 2223 | 0 | 62 | MALE | G2 | Stage III | T3 | N0 | M0 |
| TCGA-CW-5587 | 2226 | 0 | 62 | FEMALE | G2 | Stage III | T3 | N0 | M0 |
| TCGA-BP-5198 | 603 | 0 | 72 | MALE | G3 | Stage III | T3 | N0 | M0 |
| TCGA-B0-5116 | 657 | 0 | 52 | MALE | G3 | Stage III | T3 | N0 | M0 |
| TCGA-BP-4797 | 1107 | 0 | 34 | MALE | G3 | Stage III | T3 | N0 | M0 |
| TCGA-BP-4799 | 1133 | 1 | 70 | MALE | G3 | Stage III | T3 | N0 | M0 |
| TCGA-B0-4821 | 1230 | 1 | 68 | FEMALE | G3 | Stage III | T3 | N0 | M0 |
| TCGA-A3-3307 | 1436 | 0 | 66 | MALE | G3 | Stage III | T3 | N0 | M0 |
| TCGA-B0-5692 | 1487 | 0 | 66 | FEMALE | G3 | Stage III | T3 | N0 | M0 |
| TCGA-BP-4346 | 1493 | 1 | 57 | MALE | G3 | Stage III | T3 | N0 | M0 |
| TCGA-BP-4345 | 1516 | 0 | 62 | MALE | G3 | Stage III | T3 | N0 | M0 |
| TCGA-CZ-4863 | 1691 | 0 | 51 | FEMALE | G3 | Stage III | T3 | N0 | M0 |
| TCGA-B0-5713 | 1865 | 0 | 75 | FEMALE | G3 | Stage III | T3 | N0 | M0 |
| TCGA-B0-4827 | 885 | 1 | 77 | FEMALE | G4 | Stage III | T3 | N0 | M0 |
| TCGA-B0-5400 | 1132 | 0 | 59 | FEMALE | G4 | Stage III | T3 | N0 | M0 |
| TCGA-B0-5701 | 1732 | 0 | 65 | MALE | G4 | Stage III | T3 | N0 | M0 |
| TCGA-B0-4817 | 1019 | 1 | 81 | MALE | G3 | Stage III | T3 | N0 | M0 |
| TCGA-CJ-4891 | 819 | 1 | 57 | FEMALE | G4 | Stage III | T3 | N0 | M0 |
| TCGA-B4-5377 | 365 | 0 | 68 | FEMALE | G3 | Stage IV | T3 | N0 | M1 |
| TCGA-B0-4846 | 1200 | 1 | 52 | MALE | G2 | Stage IV | T3 | N0 | M1 |
| TCGA-CW-5591 | 2271 | 0 | 56 | MALE | G2 | Stage IV | T3 | N0 | M1 |
| TCGA-B8-4622 | 181 | 0 | 57 | MALE | G3 | Stage IV | T3 | N0 | M1 |
| TCGA-B0-4701 | 238 | 1 | 66 | FEMALE | G3 | Stage IV | T3 | N0 | M1 |
| TCGA-CJ-4644 | 336 | 1 | 48 | FEMALE | G3 | Stage IV | T3 | N0 | M1 |
| TCGA-B0-5080 | 342 | 1 | 63 | MALE | G3 | Stage IV | T3 | N0 | M1 |
| TCGA-BP-4335 | 475 | 1 | 65 | FEMALE | G3 | Stage IV | T3 | N0 | M1 |
| TCGA-CJ-4868 | 646 | 1 | 42 | MALE | G3 | Stage IV | T3 | N0 | M1 |
| TCGA-B8-4143 | 709 | 1 | 66 | FEMALE | G3 | Stage IV | T3 | N0 | M1 |
| TCGA-CZ-4857 | 1432 | 1 | 56 | MALE | G3 | Stage IV | T3 | N0 | M1 |
| TCGA-CJ-4904 | 1792 | 0 | 60 | FEMALE | G3 | Stage IV | T3 | N0 | M1 |
| TCGA-CJ-4918 | 93 | 1 | 64 | MALE | G4 | Stage IV | T3 | N0 | M1 |
| TCGA-BP-4771 | 162 | 1 | 62 | MALE | G4 | Stage IV | T3 | N0 | M1 |
| TCGA-B0-4703 | 182 | 1 | 51 | MALE | G4 | Stage IV | T3 | N0 | M1 |
| TCGA-BP-4974 | 211 | 1 | 58 | MALE | G4 | Stage IV | T3 | N0 | M1 |
| TCGA-CJ-6033 | 224 | 1 | 54 | FEMALE | G4 | Stage IV | T3 | N0 | M1 |
| TCGA-BP-4787 | 480 | 1 | 59 | FEMALE | G4 | Stage IV | T3 | N0 | M1 |
| TCGA-CJ-4890 | 2085 | 0 | 72 | MALE | G4 | Stage IV | T3 | N0 | M1 |
| TCGA-B0-5094 | 333 | 1 | 62 | MALE | G2 | Stage IV | T3 | N0 | M1 |
| TCGA-CW-5585 | 2609 | 0 | 51 | MALE | G2 | Stage IV | T3 | N0 | M1 |
| TCGA-BP-4352 | 344 | 1 | 74 | FEMALE | G4 | Stage IV | T3 | N0 | M1 |
| TCGA-BP-5201 | 951 | 0 | 63 | MALE | G4 | Stage IV | T3 | N0 | M1 |
| TCGA-DV-A4VX | 1626 | 1 | 59 | MALE | G4 | Stage IV | T3 | N0 | M1 |
| TCGA-BP-4798 | 334 | 1 | 74 | MALE | G4 | Stage IV | T3 | N0 | M1 |
| TCGA-BP-4770 | 329 | 1 | 73 | FEMALE | G4 | Stage IV | T4 | N0 | M0 |
| TCGA-B0-4690 | 43 | 1 | 65 | MALE | G3 | Stage IV | T4 | N0 | M1 |
| TCGA-B0-4814 | 168 | 1 | 58 | MALE | G3 | Stage IV | T4 | N0 | M1 |
| TCGA-B0-4688 | 101 | 1 | 46 | MALE | G4 | Stage IV | T4 | N0 | M1 |
| TCGA-B0-4699 | 110 | 1 | 74 | MALE | G4 | Stage IV | T4 | N0 | M1 |
| TCGA-BP-4970 | 433 | 0 | 44 | MALE | G3 | Stage III | T1 | N1 | M0 |
| TCGA-A3-3347 | 1610 | 1 | 76 | FEMALE | G2 | Stage III | T1 | N1 | M0 |
| TCGA-CJ-4869 | 2554 | 0 | 49 | MALE | G2 | Stage III | T2 | N1 | M0 |
| TCGA-B4-5838 | 166 | 0 | 52 | MALE | G2 | Stage III | T3 | N1 | M0 |
| TCGA-B0-4810 | 478 | 1 | 47 | MALE | G3 | Stage III | T3 | N1 | M0 |
| TCGA-AK-3426 | 885 | 1 | 37 | MALE | G3 | Stage III | T3 | N1 | M0 |
| TCGA-BP-4761 | 182 | 0 | 57 | MALE | G4 | Stage III | T3 | N1 | M0 |
| TCGA-B8-5158 | 293 | 0 | 56 | MALE | G4 | Stage III | T3 | N1 | M0 |
| TCGA-CW-5584 | 164 | 1 | 74 | MALE | G3 | Stage III | T3 | N1 | M0 |
| TCGA-AK-3430 | 480 | 1 | 61 | MALE | G3 | Stage III | T3 | N1 | M0 |
| TCGA-B0-5109 | 587 | 1 | 69 | MALE | G4 | Stage III | T3 | N1 | M0 |
| TCGA-B0-5084 | 222 | 1 | 33 | MALE | G3 | Stage IV | T3 | N1 | M1 |
| TCGA-CW-6087 | 41 | 1 | 61 | MALE | G4 | Stage IV | T3 | N1 | M1 |
| TCGA-CJ-4638 | 431 | 1 | 46 | FEMALE | G4 | Stage IV | T3 | N1 | M1 |
| TCGA-BP-4354 | 1034 | 1 | 40 | MALE | G4 | Stage IV | T4 | N1 | M1 |
| TCGA-CJ-4900 | 1714 | 1 | 69 | FEMALE | G4 | Stage IV | T4 | N1 | M1 |
